# Stability of Nano and Micro Particle Suspensions in Electrolyte Solutions: A Comparative Classical Density Functional Theory and Measurement of Force and Structure in Confined Complex Fluid

**DOI:** 10.1021/acs.jpcb.6c00681

**Published:** 2026-05-12

**Authors:** Simone Riva, Michael Ludwig, Regine von Klitzing, Ofer Manor

**Affiliations:** † Department of Chemical Engineering, 26747 Technion—Israel Institute of Technology, Haifa 3200003, Israel; ‡ Department of Physics, Technische Universität Darmstadt, Darmstadt 64289, Germany

## Abstract

We compare theory to experiment to study the suitability of different classical density functional theory (DFT) strategies to predict interactions and stability in different-size colloid mixtures in an electrolyte solution. Atomic force microscopy (AFM) measures the interaction force between a spherical silica microcolloid and a flat silica substrate across a suspension of silica nanoparticles in a solution of sodium chloride. The calculated and measured oscillatory structural and repulsive electrostatic forces give insight on the stability of microcolloids in suspensions of nanoparticles relevant to biological liquids, e.g., blood, plasma, and milk, as well as to particulate suspensions of broad size distributions. We examine a simple uniform-weight hard-sphere functional and the more advanced fundamental measure theory (FMT) functional to capture the contribution of the nanoparticle finite (excluded) volume to the free energy of the system. The two functionals reproduce the experimental force oscillation amplitudes over the nanoparticle and salt concentration range examined to a reasonable extent. FMT additionally captures the experimental scaling of the force oscillation wavelength. We further introduce internanoparticle electrostatic repulsion as a “soft” potential superimposed on the hard-sphere potential and compare this classical approach to defining an effective nanoparticle hard-sphere diameter based on the “hard” and “soft” interactions using the nonideal gas theory. The latter approach significantly simplifies the numerical implementation of the problem and compares well with experiment. Additionally, we capture the electrostatic interactions across the suspension between the microcolloid and the flat substrate by using the jellium approximation and discuss the fundamental assumptions made in this approach.

## Graphical abstract

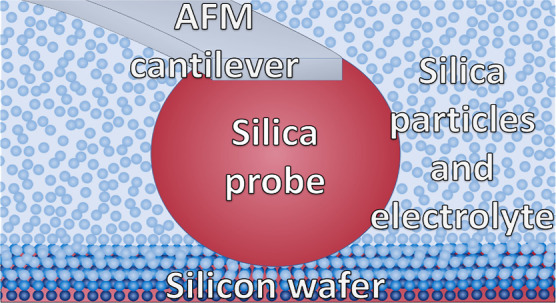

## Introduction

Predicting the microscopic structure and thermodynamic properties of inhomogeneous liquids is a long-standing problem in fluid,^1^ colloid,^2^ and interface^3^ science. Equilibrium homogeneous liquids are understood through classical thermodynamics and basic statistical mechanics. Inhomogeneous liquids of spatially nonuniform density, e.g., complex liquids consisting of different-sized molecules and colloids,^4^ interfaces,^5^ and three phase contact lines^6^ and wetting,^7^ require refined methods that capture liquid density variations and their contributions to macroscopic thermodynamic properties.^8–10^ Moreover, the problem of confined liquids, in particular delimited by two surfaces, is common in experimental colloid and interface science for studying solvation and other interaction forces between surfaces at proximity that arise due to an inhomogeneous structure of the confined solvent and electrolyte,^11^ suspended colloids,^12^ or dissolved polymers^13^ therein. A statistical treatment of the solvent density variations is required to simulate such interactions. Classical density functional theory (cDFT)^14,15^ is advantageous for investigating the microscopic structure of an inhomogeneous liquid when one wishes to convert the structure of the liquid to corresponding macro-scale thermodynamical properties, e.g., a disjoining pressure to appear in the confined liquid as a result of density variations. The simulation may then be directly compared with a corresponding macro-scale laboratory measurement that captures the force on the boundaries of the confined liquid, like force measurements that employ atomic force microscopy^16,17^ and the surface force apparatus.^18^

A number of measurements of the disjoining pressure in particulate liquids confined between opposite surfaces have been conducted to investigate solvation interactions and stabilization by a complex solvent. The liquid may be confined in a linear film geometry when the two delimiting surfaces are flat parallel plates, or a curved film when, e.g., the confinement is between a microcolloid and a flat surface, two microcolloids, or two cylindrical bodies customary in surface force apparatus measurements. Such measurements demonstrate the stabilization of a microcolloid dispersion containing various nanoparticles (NPs)^19^—whether neutral, e.g., nonionic micelles,^20^ or charged, e.g., anionic surfactant micelles,^21,22^ colloidal dispersions that support the formation of nanogels,^23^ and polyelectrolite dispersions.^13,24,25^

The presence of nanoparticles (NPs) in a confined film supports a structural interaction force, a force oscillating on the length scale of the NP size, on the boundaries of the confined suspension.^26–29^ The oscillatory interaction force measured in experiments is a product of the suspended NP layering between the confining surfaces. Upon surface approach, the NP layering results in alternate structural repulsive force maxima and depletion attractive minima when the ordered NP layers parallel to the surfaces are compressed and expelled, respectively. The hard core (excluded volume and other repulsive interactions) of the pressed layers of NPs in the confined film resists film thinning. However, as the film is further compressed, eventually one layer is expelled from the confined film to the bulk of the liquid in contact with the edges of the confinement to result in the release of stress. The presence of several layers of NPs to appear in the confined liquid film between microcolloids in a suspension supports an array of repulsive energy barriers to the microcolloid coagulation. As a consequence, it increases the stability of the intervening film between colloids and therefore the stability of a suspension of microcolloids that undergo collisions in complex NP suspensions.

Starting from the 1980s, several studies used cDFT to solve the problem of inhomogeneous liquid structures near a delimiting surface. This was done under the weighted density approximation (WDA), to which many different approaches were developed. The main strategies modeled inhomogeneous liquids as hard sphere (HS) fluids: Tarazona and Evans^30^ developed a free energy functional focused on reproducing the correct short-range HS correlations, which better capture the properties of the HS fluid next to a wall, rather than the long-range fluctuations. This was done by means of a weight function which is constant inside a sphere of radius equal to the HS diameter and null elsewhere. This approach provided reliable results at low HS concentrations, since it is based on the dilute approximation of the direct correlation function. Shortly after, Tarazona^31^ developed a semiempirical but more precise density-dependent weight function which better reproduces the separation between HS layers in proximity of a wall. This procedure was extrapolated to give correct results at greater densities than before. Tan et al.^32^ further extended Tarazona’s density-dependent weight function to study HS mixtures and tested the obtained density profiles against Monte Carlo simulations. These analyses were commonly compared to numerical simulations for validation^10,33,34^ rather than to experiment because of the restricted number of corresponding experiments available at the time. Several years later, Rosenfeld^35,36^ introduced a functional based on the decomposition of the HS Mayer function for the many-body problem to individual-sphere fundamental measures, i.e., fundamental measure theory (FMT), which improved the precision of DFT calculations up to rather high liquid densities.^37,38^ The approach was applied to calculate density structures of HS mixtures near hard walls^5^ and the structural interaction between large HSs in a fluid of smaller spheres^39^ and was validated using molecular dynamics^40^ and Monte Carlo^41,42^ simulations.

A DFT was also developed for the calculation of the potential between colloids to extend the solvent from a HS liquid to a polymer solution.^43^ However, polymers were described as chains of hard monomers via the free energy from the first-order thermodynamic perturbation theory and the same weight function as in the work of Tarazona and Evans; the analysis remained limited to hard-sphere excluded-volume interactions. In the same study, the potential was calculated using integral equations and the Percus–Yevick closure.^44^ A similar study employed the Rosenfeld FMT rather than the Tarazona and Evans weight function.^45^ Forsman and Woodward^46,47^ further studied polymer-induced depletion forces between hard spheres by means of DFT and compared them to the Asakura–Oosawa model. This was done for ideal polymers (i.e., that can penetrate each other but not the hard colloids) and for a Lennard-Jones (LJ) monomer–monomer potential. When accounting for the finite size of monomers, their excluded volume was included using a coarse-grained density as an entropic penalty in the free energy. Enthalpy was constituted by a mean-field corrective term for the LJ interactions in the density functional. This mean-field approximation does not account for particle–particle correlations and thus cannot reproduce large density oscillations. It is known for underestimating the particulate structure^48^ and it is suitable to describe short-range monotonic attractive depletion (or an extremely weak repulsive peak) similar to the Asakura–Oosawa potential, but not structural interactions. In fact, no oscillations associated with particle layering were observed. Colloids were also represented as charged spheres^49^ with electrical double layer interactions calculated via the Poisson–Boltzmann theory, which is independent of the depletion force.

The first HS models to provide analytical expressions for structural forces due to excluded volume by the presence of spheres were derived based on the Percus–Yevick theory. Henderson^50^ found an analytical expression for the Laplace transform of the interaction energy between large spheres in a fluid of smaller HSs and inverted it with the aid of numerical computations to obtain the interaction force between the large spheres. Trokhymchuk et al.^51^ inverted the same Laplace transform asymptotically for large film thicknesses to obtain an analytical expression depending on numerically evaluated parameters. However, similarly to the aforementioned DFTs, these semianalytical theories following the Percus–Yevick approximation were limited to HS models, which capture the behavior of neutral NPs with no relevant interactions besides their volume exclusion. An example where such theory simulated experiment well is a colloidal-probe atomic force microscopy (AFM) measurement of nonionic micelles.^20^

Experiments conducted with charged NPs, involving electrostatic interactions in confined suspensions, typically employ empirical expressions for the analysis of their results. A general expression of the structural force, namely an exponentially decaying harmonic function,^52^ was borrowed from the HS models and applied using a number of parameters fitted from AFM data to model successfully several experiments that further involved electrical double layer (EDL) interactions, e.g., Tabor et al.,^19^ Schön et al.,^23^ Qu et al.,^24^ Klapp et al.,^26,27^ Zeng et al.,^28^ Ludwig et al.,^29^ Zeng and von Klitzing.^53^ Other AFM force measurements were tested against Monte Carlo simulations.^54,55^ Moreover, previous approaches introduced HS models to capture oscillatory forces to result from charged NPs in electrolyte solutions using an effective NP radius. The effective radius is related to the volume excluded by the NP by its “hard” nondeformable core and by the “soft” EDL repulsion between NPs. The effective NP size was estimated as the sum of the nominal NP radius (associated with the occupied solid volume of the particle) and the Debye length of the EDL around the NP.^56,57^ However, this approach was shown to be applicable only for specific experimental conditions^53^ rather than as a general strategy.

In the aforementioned experiments on charged NPs, the latter were immersed in electrolyte solutions. Here, EDLs appear not only around the charged NPs, but also next to the charged confining surfaces. Hence, EDL interactions between the confining surfaces across a suspension of charged NPs require special care: it is experimentally challenging to untangle the contributions to the force on the confining surfaces from the EDL interaction across the charged NP suspension and from the excluded volume of the latter, i.e., from the structural interaction.^58^ The measurement gives the total stress, which to leading order is the sum of structural and EDL stresses. However, for sufficiently large volume excluded by the NPs, the confinement and their sheer size renders them immobile compared with the ion species. As we will show, theory may thus decouple the problems for the distribution of NPs and of ions in the EDL to the superposition of two separate functionals by means of the *jellium* approximation.^58^ Using this approximation, we include the entropy and excluded volume of NPs in a hard-sphere functional to obtain the main contributions to their spatial distribution under confinement. The leading-order contribution of NPs to the electric field appears in another functional for the electrostatic problem as a spatially uniform charge, ignoring their charge distribution. Hence, under the *jellium* approximation, the screening of the electrostatic potential generated by the charged confining surfaces is an effect of the small ions alone, rendering the EDL problem mathematically decoupled from the confined NP distribution problem. We will give more details in the next section.

The EDLs and their interaction are described by a functional developed by Mier-y-Teran et al.,^59^ that in the ideal case translates to the classic Poisson–Boltzmann theory, to which we add the *jellium* uniform charge. This approach is consistent with our DFT strategy and enables the inclusion of interactions beyond the ideal Poisson–Boltzmann model. We investigated contributions from the ion excluded-volume interactions, which were found negligible at the concentrations under exam.^60^

DFT, despite being effective in structural calculations, has been used to capture the structure of hard spheres next to a hard wall and only in one case to our knowledge to predict an oscillatory potential between large colloids by nanoparticles,^39^ going beyond mean-field calculations of monotonic depletion by polymers.^46,47,49^ As a consequence, it is not clear which DFT formulation is better at modeling real measurements of structural forces in nanoparticle suspensions. Moreover, DFT enables a consistent treatment of the structural and EDL forces, which are entangled in measurements and are usually modeled with different theories, a feature that is not fully taken advantage of.

Here we use classical DFT to reproduce measurements of structural and EDL forces in NP suspensions in electrolyte and assess the effectiveness of different approximations adopted for the free energy functional. We add the soft tail of varying EDL interactions to the most well-known HS models and consider EDL interactions between the confined surfaces across the charged NP suspension. We show that DFT may be employed self-consistently to reproduce measurements of structural force curves and develop a general model valid for ionic suspensions, which accounts for EDL repulsion between like-charged NPs, that was not present in previous theories. The theory, devoid of fitting parameters, agrees well with experiment with judicial choice of HS models and integration of EDL interactions in the overall framework.

We introduce three weighting approximations for the DFT of HSs—namely Tarazona and Evans’ simple weight, Tarazona’s density-dependent system of weights, and FMT—and compare the results to AFM experiments on charged silica NP suspensions in the presence of added NaCl.^58^ We advance beyond the pure hard-core theories to model interactions between charged NPs by two methods, namely introducing an effective HS diameter, a nonlinear function of the Debye length and charge on the NPs, and using a functional that includes the free energy of hard-core and “soft” EDL interactions between NPs.

We compare our theoretical prediction of force curves to experiment,^58^ sketched in Figure 1, to validate different modeling strategies. In the experiment, a spherical silica particle of 5 μm diameter was used as a probe, glued to the rectangular AFM cantilever to measures the interaction force with a smooth silica surface. The system was immersed in a suspension of negatively charged silica NPs of *d*_NP_ = 15.8 ± 2.9 nm diameter in an aqueous solution of sodium chloride, NaCl. The ζ-potential at the surface of the colloidal probe and flat substrate was measured to be ψ_0_ = −28.6 ± 2.9 mV and kept approximately constant under the different conditions of the experiment. The NPs were present in three volume fractions: $\phi \equiv \pi d_{NP}^{3}n_{3}^{b}/6=0.0061,0.031,0.065$ , where *n*_3_^*b*^ is the bulk density of NPs per volume. The charge on the NPs, to result from the release of sodium counterions, (Na^+^), from the NP surface to the solution, was found to vary with the NP concentration from the counterion concentration measurement. The total ionic strength of the suspension was determined via conductivity measures for different concentrations, *c*(NaCl) of added NaCl salt. The conductivity grew linearly with the added salt concentration, independently of the NP volume fraction, indicating that the salt is fully dissociated at all NP concentrations. The ionic strength measured in the absence of added salt was associated with the concentration of counterions neutralizing the NPs, *c*_0_(Na^+^), which was determined as 1.98 ± 0.08 mM at ϕ = 0.0061, 6.56 ± 0.12 mM at ϕ = 0.031 and 12.16 ± 0.12 mM at ϕ = 0.065. The total ionic strength was given by

1 $$I=\frac{c_{0}\left({Na}^{+}\right)}{2}+c\left(NaCl\right)=\frac{n_{1}^{b}+n_{2}^{b}}{2N_{A}}$$

where *n*_1_^*b*^ and *n*_2_^*b*^ are the bulk number density (bulk concentration) of the Na^+^ and Cl^–^ ions, respectively, and *N*_A_ is the Avogadro number. The amount of added salt was 0.1, 1, or 5 mM. The curve of the force on the colloidal probe, *F*, versus the minimum separation between the probe and the substrate, *h*, was obtained by moving the AFM cantilever toward the substrate at a rate of 100 nm/s, and averaging 30 repeating measurements.

**Figure 1:**
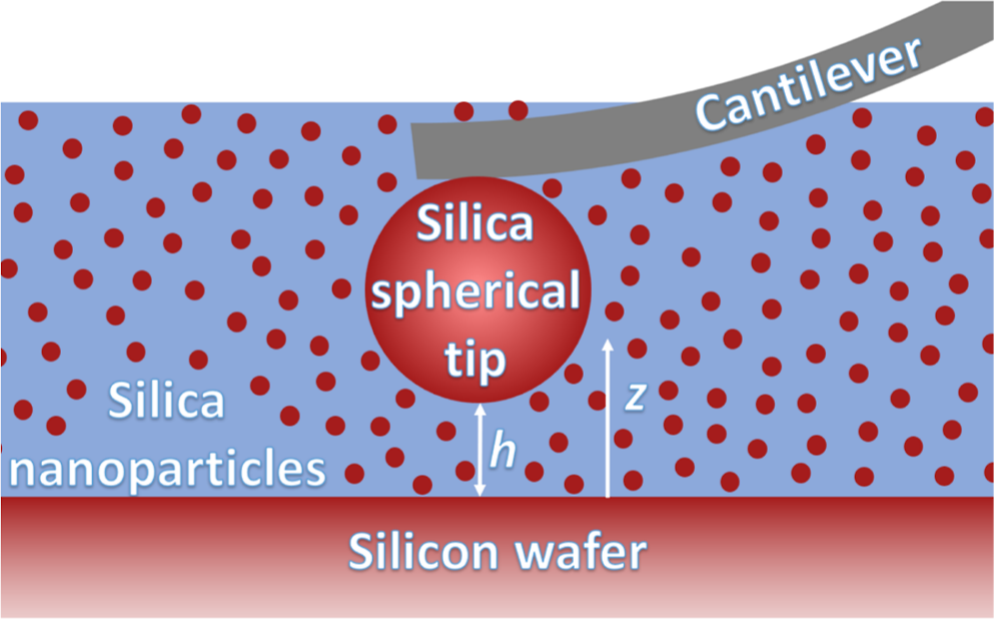
Scheme of the AFM measurement of the force between a silica colloidal probe and a silicon wafer with an upper flat silica surface across a suspension of negatively charged silica NPs in electrolyte.

In the experiment, NPs did not behave as simple HSs. Measurements revealed different oscillatory force profiles when the salt concentration is varied at fixed NP volume fractions as a consequence of the modified screening of the EDL interactions between the NPs. In particular, the oscillation amplitude decreased when adding salt.

## Theoretical Methods

### General Formulation

Different to classical statistical thermodynamics, cDFT is a continuum theory. It satisfies variational minimization of a free-energy density functional to obtain the equilibrium properties of classical inhomogeneous and particulate fluids.^8,15^ In most problems involving inhomogeneous liquid films, the grand potential Ω = *F* −∑_*i*_μ_*i*_ *N*_*i*_ = *U* – *TS* – ∑_*i*_μ_*i*_ *N*_*i*_ is the relevant thermodynamic potential, where *U* and *F* are the internal energy and Helmholtz free energy, respectively, *T* and *S* are the temperature and entropy, respectively, and μ_*i*_ and *N*_*i*_ are the chemical potential and number of molecules of species *i*, respectively. Its differential, dΩ = – *P*d*V* – *S*d*T* – ∑_*i*_ *N*_*i*_dμ_*i*_, is useful for assessing the free energy variation of a system in contact with a reservoir at fixed temperature *T*, volume *V*, and chemical potentials μ_*i*_.

In Supporting Information, we provide the grand potential functional for a one-component system. Considering the experiment at hand, where the NPs are in an electrolyte solution of the univalent sodium chloride salt, the free energy is a function of the sodium ion density, *n*_1_, chloride ion density, *n*_2_, and NP density *n*_3_. The corresponding grand potential is^37^

2 $$\Omega \left[\{n_{i}\}\right]=F\left[\{n_{i}\}\right]-\sum_{i}\mu_{i}\int drn_{i}\left(r\right)+\sum_{i}\int drn_{i}\left(r\right)V_{ext,i}\left(r\right)$$

where *i* = 1, 2, 3. Here the first term on the right-hand side (r.h.s.) of the equation, $F\left[\{n_{i}\}\right]$ , is the intrinsic Helmholtz free energy, defined in the absence of an external field, the second is the previous term containing the chemical potentials, −∑_*i*_μ_*i*_ *N*_*i*_, and the last term accounts for the external potential for each species, *V*_ext,*i*_(***r***). Moreover, ∫d ***r*** represents a volume integral. The functional is variationally minimized to determine the equilibrium densities, *n*_*i*,eq_(***r***), which vary in space; at these equilibrium densities the grand potential functional corresponds to the grand potential of the system, Ω. The Euler–Lagrange equations and the grand potential of the system are therefore

3 $${\frac{\delta \Omega \left[\{n_{i}\}\right]}{\delta n_{i}\left(r\right)}|}_{n_{i}=n_{i,eq}}={\frac{\delta F\left[\{n_{i}\}\right]}{\delta n_{i}\left(r\right)}-\mu_{i}+V_{ext,i}\left(r\right)|}_{n_{i}=n_{i,eq}}=0,\left(i=1,\;2,\;3\right),,\Omega \left[\{n_{i}=n_{i,eq}\}\right]=\Omega$$

The chemical potentials—equal at equilibrium in the confined particulate liquid and in the bulk reservoir—are usually evaluated by applying the three equations in the first line to the bulk homogeneous suspension, where the constant bulk densities are given by *n*_*i*_(***r***) = *n*_*i*_^*b*^ and the external potential vanishes, i.e., *V*_ext,*i*_(***r***) = 0, so that $\mu_{i}={\delta F\left[\{n_{i}\}\right]/\delta n_{i}\left(r\right)|}_{n_{i}=n_{i}^{b}}$ .

Moreover, the radius of the AFM spherical probe is *R* = 2.5 μm, and thus much larger than the separations between its tip and the substrate at which the force is measured, *h* ∼ 10–100 nm. Thus, we may use the Derjaguin approximation to consider the interaction energy per area, *W*(*h*), associated with an equivalent confinement by two flat parallel surfaces and relate it to the force experienced by the spherical probe, *F*(*h*) = 2π*RW*(*h*), through the geometrical coefficient 2π*R*. This is achieved by placing planar boundaries at *z* = 0 and *z* = *h*, *h* being the minimum separation between the surfaces of the substrate and the colloidal probe in the AFM experiment. The problem is thus rendered one-dimensional and solely dependent on the transverse coordinate to the flat surfaces, *z*, i.e., *n*_*i*_(***r***) = *n*_*i*_(*z*).

Outside of these boundaries, the density is kept equal to a value corresponding to the maximum packing fraction of hard spheres in computations to reproduce the behavior of hard walls: the excess free energy term due to volume exclusion that we will include in the functional diverges at *z* = 0 and *z* = *h* because of the high packing level inside the walls, thus not allowing the presence of NPs and effectively creating an exclusion layer with density vanishing at *z* = 0 and *z* = *h*. This wall effect fades within a distance of one NP diameter from the surfaces and the density is found to become significant already at a distance of one NP radius. Allowing the density to vary freely in the interval 0 < *z* < *h* subject to these constraints, we attempt to produce realistic values close to contact with the walls.

The functional Ω­[{*n*_*i*_}] is minimized by solving the three eq 3 to obtain the equilibrium density distributions *n*_*i*,eq_(*z*) (*i* = 1, 2, 3), at different separations, *h*, between the substrate and the tip of the AFM colloidal probe. The equilibrium densities are then used to calculate the grand potential, Ω, for each value of separation, *h*. The interaction energy per area, *A*, is given by

4 $$W\left(h\right)\equiv \Omega \left(h\right)/A-\Omega^{b}/V\times h-2E_{s}/A$$

The interaction energy, *W*(*h*), vanishes at large separations, *h* → ∞, where the confining flat surfaces do not interact. Hence, it is constituted by the grand potential per unit area, Ω­(*h*)/*A*, against the reference free energy, which is not associated with the interaction. The reference free energy is given by the sum of two contributions: one contribution is the grand potential per unit area of the suspension at bulk conditions across the separation *h*, but in the absence of the confining surfaces. This contribution increases linearly with *h* since the volume between the confining surfaces where the interaction takes place increases at the expense of the volume of bulk suspension outside the interaction region. It is thus given by the bulk energy density

5 $$\Omega^{b}/V=\Omega \left[\{n_{i}^{b}\}\right]/V=F\left[\{n_{i}^{b}\}\right]/V-\sum_{i}\mu_{i}n_{i}^{b}$$

multiplied by *h*. This expression derives from eq 2, where we assume the absence of the external potential in the bulk, constant densities *n*_*i*_^*b*^ and *V* = ∫d ***r***. The second contribution is the surface energy *E*_s_ per area, *A*, of the two surfaces at large separations, associated with the structures (NP layers and ionic EDLs) that the suspension forms next to the isolated surfaces. This quantity is obtained per confining surface by calculating the free energy of a flat surface neighboring a half-space of the NP suspension in the electrolyte solution.

The main challenge remaining is the identification of the intrinsic Helmholtz free energy functional, $F\left[\{n_{i}\}\right]=F_{id}\left[\{n_{i}\}\right]+F_{ex}\left[\{n_{i}\}\right]$ , which is comprised of an ideal and excess components, $F_{\mathrm{id}}$ and $F_{\mathrm{ex}}$ , respectively. The ideal contribution is associated with the entropy of an ideal gas mixture

6 $$F_{id}\left[\{n_{i}\}\right]=k_{B}T\sum_{i}\int drn_{i}\left(r\right)\left[\log\left(d_{NP}^{3}n_{i}\left(r\right)\right)-1\right]$$

where for scaling length we use the nominal diameter of NPs (*d*_NP_), which is a characteristic length in the experiment, rather than the commonly used thermal de Broglie wavelength.

Capturing realistic systems further requires introducing the excess contribution to the Helmholtz free energy, *F*_ex_, which is specific for each system under investigation. In the current problem we look for contributions to *F*_ex_ from the repulsion between NPs, associated with the NP hard core and the repulsive inter-NP EDL interaction, and from the EDL interaction between the confining surfaces across the charged NP suspension in electrolyte solution. To simplify the problem, we first consider the contribution to Ω from the NP arrangement, Ω_NP_, which produces the structural force. We use the *jellium* approximation to decouple the NP problem from the EDL problem, so that

7 $$\Omega \left(n_{1},n_{2},n_{3}\right)\approx \Omega_{NP}\left(n_{3}\right)+\Omega_{EDL}\left(n_{1},n_{2}\right)$$

As noted in the introduction, the *jellium* approximation consists in considering the NPs as a source of a uniform background charge, which corresponds to the NP bulk density, when treating the distributions of ions in the electrolyte solution: ions support the diffusive layer of the EDL and contribute to the electrostatic screening. NPs carry a much greater charge than ions, which might be expected to increase the ionic strength in the electrolyte solution. However, due to their large size and spacing, the NPs do not contribute to the Debye length, namely the screening length of the electric potential. The average distance between NPs at their bulk concentration is 30–70 nm, namely 1 order of magnitude greater than the Debye length (3–9 nm when solely considering ion contribution). Thus, the ion spatial distribution near the charged confining surfaces or near the surface of a charged NP is not sensitive to the specific distribution of other NPs that are further away at distances greater than a Debye length. Moreover, even in the case of strong confinement by the two surfaces, the NPs are arranged in ordered layers, as proved by the experimental observation of large oscillatory forces, which makes their distribution uniform on the length scale of the electrostatic potential screening. In fact, the NP nominal size of 15.8 nm is several times larger than the Debye length in the experiment, and so is their spacing. NPs arrange in space mainly due to their low density and large excluded-volume effects, rather than following the equilibrium Boltzmann distribution by reacting to the electric potential like sodium and chloride ions . However, NP charge affects ions with a mean-field excess electric potential, well approximated by the uniform *jellium* background charge.

Hence, under the *jellium* approximation we determine the EDL structure by assuming that only the ions are at equilibrium for each given separation, *h*, so that Ω_EDL_ has to be minimized only with respect to *n*_1_ and *n*_2_. The NP density, *n*_3_, is governed by Ω_NP_.

### Nanoparticle Excluded Volume

The measured structural force oscillates with separation, *h*, which is associated with the oscillation of the NP density profile with *h*. To capture the density oscillations, i.e., to describe packing effects, layering or freezing, within the realm of cDFT one may adopt the weighted density approximation (WDA).^15,30^ The formalism introduces nonlocality in the free energy to reproduce the correct particle–particle correlations and hence it is useful for capturing large density inhomogeneities. The corresponding excess Helmholtz free energy is given by

8 $$F_{ex}\left[n_{3}\right]=\int drn_{3}\left(r\right)\Psi \left({\mathrm{n̅}}_{3}\left(r\right)\right)$$

where Ψ­(*n*_3_(***r***)) is the excess free energy per particle of a local homogeneous system at density *n*_3_(***r***). The averaged density ${\mathrm{n̅}}_{3}\left(r\right)$ introduces nonlocality and is defined by

9 $${\mathrm{n̅}}_{3}\left(r\right)\equiv \int dr^{\prime }n_{3}\left(r^{\prime }\right)w\left(r-r^{\prime }\right)$$

where *w* is an integrable and normalized weight function that satisfies ∫d ***r*** *w*(***r***) = 1. The choice of the weight function, *w*, is aimed at reproducing the particle–particle direct correlation function in the system under study via the second functional derivative of $F_{ex}\left[n_{3}\right]$ .

A local density formulation of DFT would be inconsistent with the occurrence of large liquid density variations. The weighted density function, ${\mathrm{n̅}}_{3}\left(r\right)$ , is necessary to smoothen the local density, *n*_3_(***r***). In fact, the local density presents large peaks near a confining surface that could produce unrealistically large contributions to the free energy Ψ­(*n*_3_), which is a rapidly growing function of *n*_3_ In reality, density peaks are alternated with low density minima Averaging over a range comparable to the oscillation period captures the physics of these interactions and avoids numerical divergence.

The contribution to the grand potential from the excluded volume by NPs that are confined by hard walls in the WDA reads

10 $$\Omega_{NP}\left[n_{3}\right]=\int drn_{3}\left(r\right)\left\{k_{B}T\left[\ln\left(d_{NP}^{3}n_{3}\left(r\right)\right)-1\right]+\Psi \left({\mathrm{n̅}}_{3}\left(r\right)\right)\right\}-\mu_{3}\int drn_{3}\left(r\right)$$

where the first and second terms in the first integral on the r.h.s. of the equality are the ideal and excess Helmholtz energy, respectively. The latter is derived from the free energy associated with the excluded volume of the NPs in a homogeneous liquid of HSs, Ψ­(*n*_3_). In eq 10 we have neglected the external potential, *V*_ext_, since the surfaces confining the suspension are hard walls for the purpose of a HS representation of the NPs. Therefore, the walls confine the NPs within the boundaries of the suspension film, but do not affect the NPs therein through a long-range potential.

Assuming that the NPs are hard spheres (HSs), a common approximation for their excess free energy is the Carnahan–Starling equation, also used here^61^

11 $$\Psi \left(n_{3}\right)=k_{B}T\phi \frac{4-3\phi }{{\left(1-\phi \right)}^{2}}$$

in which $\phi =\frac{3}{4\pi d_{\mathrm{NP}}^{3}}$ is the volume fraction of HSs. This originates from an approximate equation of state for HSs.

### Constant Weight

The weight function *w*(***r***) is chosen to reproduce known approximations of the direct correlation function of HSs, such as the Percus–Yevick approximation.^62^ Tarazona and Evans^30^ captured short-range correlations of HSs by using the weight function

12 $$w\left(r\right)=\{\begin{array}{ll} 3/4\pi d_{NP}^{3} & \text{if}\;\left|r\right|\leq d_{NP}\\ 0 & \text{if}\;\left|r\right|>d_{NP} \end{array}$$

Thus, the NP density is spatially averaged with uniform weight inside a sphere of radius equal to the NP diameter, yielding

13 $${\mathrm{n̅}}_{3}\left(r\right)=\frac{3}{4\pi d_{NP}^{3}}\int_{\left|r-r^{\prime }\right|<d_{NP}}dr^{\prime }n_{3}\left(r^{\prime }\right)$$

The minimization of the grand potential functional, eq 10, with the choices detailed here for the free energy per particle and the weight function, gives

14 $$\frac{\delta \Omega_{NP}}{\delta n_{3}\left(z\right)}=k_{B}T\;\ln\left(d_{NP}^{3}n_{3}\left(z\right)\right)+\Psi \left({\mathrm{n̅}}_{3}\left(z\right)\right)+\frac{3}{4\pi d_{NP}^{3}}\int_{\left|r-r^{\prime }\right|\leq d_{NP}}dr^{\prime }n_{3}\left(z^{\prime }\right)\Psi^{\prime }\left({\mathrm{n̅}}_{3}\left(z^{\prime }\right)\right)-\mu_{3}=0$$

where the chemical potential is $\mu_{3}=k_{B}T\;\ln\left(d_{NP}^{3}n_{3}^{b}\right)+\Psi \left(n_{3}^{b}\right)+n_{3}^{b}\Psi^{\prime }\left(n_{3}^{b}\right)$ and Ψ′ is the derivative of Ψ.

The theory, appropriate for small HS densities, was extended by Tarazona who added a correction for greater HS densities using a density-dependent weight function.^31^ However, this correction to the uniform-weight theory is significantly more demanding computation-wise. Convergence of solutions was found to be challenging when it comes to simulating the problem at hand that involves hard spheres confined by two opposing solid surfaces.

### Fundamental Measure Theory (FMT)

Instead, we employ the fundamental measure theory (FMT) developed by Rosenfeld^35,36^ for medium to large HS densities. The theory relies on six weight functions ω^(α)^(***r***), where α takes four values for scalar functions (0, 1, 2 and 3) and two for vectorial functions (1 and 2), see Supporting Information for more details. Each of these is associated with a weighted density

15 $$n^{\left(\alpha \right)}\left(r\right)=\int dr^{\prime }n_{3}\left(r^{\prime }\right)\omega^{\left(\alpha \right)}\left(r-r^{\prime }\right)$$

The minimizing equation in this case is

16 $$\frac{\delta \Omega_{NP}}{\delta n_{3}\left(r\right)}=k_{B}T\;\ln\left(n_{3}\left(r\right)/n_{3}^{b}\right)+\sum_{\alpha }\int dr^{\prime }\left\{\frac{\partial \Phi \left(\{n^{\left(\alpha \right)}\left(r^{\prime }\right)\}\right)}{\partial n^{\left(\alpha \right)}\left(r^{\prime }\right)}-{\frac{\partial \Phi \left(\{n^{\left(\alpha \right)}\left(r^{\prime }\right)\}\right)}{\partial n^{\left(\alpha \right)}\left(r^{\prime }\right)}|}_{n_{3}=n_{3}^{b}}\right\}\frac{\delta n^{\left(\alpha \right)}\left(r^{\prime }\right)}{\delta n_{3}\left(r\right)}=0$$

with the function

17 $$\frac{\Phi \left(\{n^{\left(\alpha \right)}\left(r\right)\}\right)}{k_{B}T}=-n^{\left(0\right)}\;\ln\left(1-n^{\left(3\right)}\right)+\frac{n^{\left(1\right)}n^{\left(2\right)}}{1-n^{\left(3\right)}}+\frac{1}{24\pi }\frac{{\left(n^{\left(2\right)}\right)}^{3}}{{\left(1-n^{\left(3\right)}\right)}^{2}}-\frac{n^{\left(1\right)}\cdot n^{\left(2\right)}}{1-n^{\left(3\right)}}-\frac{1}{8\pi }\frac{n^{\left(2\right)}\left(n^{\left(2\right)}\cdot n^{\left(2\right)}\right)}{{\left(1-n^{\left(3\right)}\right)}^{2}}$$

We provide further insight in Supporting Information.

### Electrical Double Layer (EDL) Interactions

Besides contributions to the grand potential from the excluded volume of NPs, there are two distinct contributions from electrical double layer (EDL) interactions. One is inter-NP EDL interactions. The other is EDL interactions across the NP suspension via the electrostatic potential that originates from the charged confining surfaces and is screened by the electrolyte.

### Internanoparticle Interactions

In our theory we applied the Tarazona and Evans functional for low concentrations and FMT for high concentrations after modifying them as follows. The HS theory is already suitable for noncharged NPs, such as nonionic micelles,^20^ for which it is only necessary to solve eqs 14 or 16, with *d*_NP_, the nominal NP diameter. Since we are interested in comparing our results to experiments on charged silica NPs,^58^ we model these as HSs with an effective diameter larger than their nominal size to account for the additional inter-NP electrostatic repulsion. This is not done by simply adding the Debye length of the surrounding EDL to the NP nominal radius, which was attempted before but proved to be rather arbitrary and not consistent at all volume fractions. Instead, we consider the total integral of the Mayer function of the HS and EDL interparticle interactions through the second virial coefficient. In a pure HS liquid, the latter is closely related to the volume of the spheres, being half of the volume made inaccessible for the center of a particle by the presence of another particle. Since we are approximating our system with a HS model, we take the second virial coefficient of the real system as half the excluded volume of an equivalent HS system.

In the experiment the silica NPs are negatively charged following the release of sodium counterions, leading to an excess of cations over anions in the electrolyte solution. The ratio between the content of the two ions is dependent on the concentration of NPs. The charge on NPs and the ion concentration for our experimental reference was estimated previously.^58,60^

The pair-potential for the interaction between NPs is given by a hard-core volume exclusion, whose effect was discussed in the previous section, and a long “soft” tail for the EDL interaction potential

18 $$V\left(r\right)=\{\begin{array}{ll} \infty & \text{if}\;r\leq d_{NP}\\ V_{EDL}\left(r\right) & \text{if}\;r>d_{NP} \end{array}$$

We estimate the EDL interaction by^63^

19 $$V_{EDL}\left(r\right)=\pi \epsilon \frac{d_{NP}^{2}}{r}\psi_{s,NP}^{2}\;\ln\left[1+e^{-\kappa \left(r-d_{NP}\right)}\right]$$

where κ^–1^ is the Debye length of the suspension and ψ_s,NP_ is the ζ-potential on the NP surface, evaluated from measurement through their surface charge.

The second virial coefficient in the theory of nonideal gases accounts for the pair-potential between two interacting gas particles at first order in density. Treating the NPs in our problem as a nonideal gas, the corresponding second virial coefficient reads

20 $$B_{2}=2\pi \int_{0}^{\infty }drr^{2}\left[1-e^{-V\left(r\right)/k_{B}T}\right]=\frac{2\pi }{3}d_{NP}^{3}+2\pi \int_{d_{NP}}^{\infty }drr^{2}\left[1-e^{-V_{EDL}\left(r\right)/k_{B}T}\right]$$

where the first term is associated with the hard sphere (HS) interaction (with nominal diameter of the NPs *d*_NP_ = 15.8 nm from the experiment) and the second is associated with the EDL potential tail. Moreover, in the case of interactions solely due to HSs of effective diameter *d*_NP,eff_, the second virial coefficient reads

21 $$B_{2}=\frac{2\pi }{3}d_{NP,eff}^{3}$$

The analogy between [Disp-formula eq20] and 21 gives an effective NP diameter

22 $$d_{NP,eff}^{3}=d_{NP}^{3}+3\int_{d_{NP}}^{\infty }drr^{2}\left[1-e^{-V_{EDL}\left(r\right)/k_{B}T}\right]$$

that accounts for both the contribution to the inter-NP pair potential from the NP hard core and soft EDL interactions.

To model charged NPs, we substitute *d*_NP,eff_ to *d*_NP_ in the contribution to the grand potential from the exclusion volume by NPs, i.e., in the second term (excess term) of eq 10, where the diameter appears implicitly as discussed in the previous section, whether it is calculated with the constant weight or FMT. This reduces the numerical complexity associated with capturing inter-NP interactions in our problem. The simplified approach in [Disp-formula eq22] facilitates stability in a wide range of numerical solutions for our problem as will be shown later.

A similar approach was introduced by Barker and Henderson^64^ in their perturbation theory for liquids to approximate the repulsive part of a general potential as an equivalent hard-sphere potential with an effective diameter. This method proved effective in several situations, especially to model the Lennard-Jones potential and calculate thermodynamic quantities such as pressure and internal energy, as well as phase diagrams of gases.^65,66^ However, in that case the effective diameter was defined slightly differently, by a linear integral of the Mayer function, $1-e^{-V\left(r\right)/k_{B}T}$ , up to the distance where the pair potential vanishes (infinity in the case of a purely repulsive potential). Instead, we assume a three-dimensional integral in order to capture the exact virial expansion of the equation of state up to the first order.

We compare the above approach to deal with the problem of long-range inter-NP interactions to the classical strategy of introducing them as a “soft” potential contribution in the functional. Following the Tarazona and Evans method for mean-field treatment of pair-potentials, comprising hard spheres and additional “soft” interaction energy,^30^ we rewrite eq 10 as

23 $$\Omega_{\mathrm{NP}}\left[n_{3}\right]=\int drn_{3}\left(r\right)\left\{k_{B}T\left[\ln\left(d_{NP}^{3}n_{3}\left(r\right)\right)-1\right]+\Psi \left({\mathrm{n̅}}_{3}\left(r\right)\right)\right\}+\frac{1}{2}\int dr\int dr^{\prime }n_{3}\left(r\right)n_{3}\left(r^{\prime }\right)V_{EDL}\left(|r-r^{\prime }|\right)-\mu \int drn_{3}\left(r\right)$$

with *V*_EDL_(*r*) given as in eq 19. It follows that minimizing the free energy for the relevant dimension, *z*, gives

24 $$\frac{\delta \Omega_{\mathrm{NP}}}{\delta n_{3}\left(z\right)}=k_{B}T\;\ln\left(d_{NP}^{3}n_{3}\left(z\right)\right)+\Psi \left({\mathrm{n̅}}_{3}\left(z\right)\right)+\frac{3}{4\pi d_{NP}^{3}}\int_{\left|r-r^{\prime }\right|\leq d_{NP}}dr^{\prime }n_{3}\left(z^{\prime }\right)\Psi^{\prime }\left({\mathrm{n̅}}_{3}\left(z^{\prime }\right)\right)+\int_{0}^{h}dz^{\prime }n_{3}\left(z^{\prime }\right)\int_{0}^{\infty }d\rho 2\pi \rho V_{EDL}\left(\sqrt{\rho^{2}+{\left(z-z^{\prime }\right)}^{2}}\right)-\mu =0$$

Since we are splitting the pair interaction potential of NPs in two parts, the EDL interaction and the HS interaction, the former should be restricted to inter-NP separations greater than one diameter, as inside the diameter the latter already accounts for the volume exclusion (infinite potential). For this reason we restrict the EDL potential to distances *r* > *d*_NP_ and define it as 0 at *r* < *d*_NP_.

### EDL Interactions across the Suspension

The two charged surfaces confining the solution experience osmotic and Maxwell stresses, which are associated with the electrical double layers (EDLs) formed across the NP suspension by the electrolyte in response to the electrical potential they generate therein. As we discussed earlier, the *jellium* approximation removes the specific NP density distribution from the EDL problem. The latter is to be solved for the distribution of anions and cations in the presence of a uniform background charge that originates from the NPs assumed to maintain the bulk concentration.

A grand potential for the electrostatic ion interactions, which is a functional of the ionic densities in the electrolyte solution but devoid of the presence of charged NPs, is given by^59^

25 $$\Omega_{EDL}\left[n_{1},n_{2}\right]=k_{B}T\sum_{i=1}^{2}\int drn_{i}\left(r\right)\left[\ln\left(d_{NP}^{3}n_{i}\left(r\right)\right)-1\right]+\frac{1}{2}\sum_{i=1}^{2}\sum_{j=1}^{2}\iint drdr^{\prime }n_{i}\left(r\right)n_{j}\left(r^{\prime }\right)\frac{q_{i}q_{j}}{4\pi \epsilon \left|r-r^{\prime }\right|}+\sum_{i=1}^{2}\int drn_{i}\left(r\right)\left[v_{i}\left(r\right)-\mu_{i}\right]$$

here *q*_1_ and *q*_2_ are the charge of sodium and chloride ions (of number densities *n*_1_ and *n*_2_), respectively, and *v*_*i*_(***r***) = *q*_*i*_ψ_ext_(***r***) + *v*_*i*_^hw^(***r***) is the sum of the external potentials in the form of electrical potential, ψ_ext_(***r***), and steric potential, *v*_*i*_^hw^(***r***), experienced by ion *i*, which are generated by the walls confining the suspension. This functional contains the entropy of ions, the interion Coulombic energy, the potential exerted by the hard walls, *v*_*i*_, and the ion chemical potential, μ_*i*_.

We now wish to add the background negative charge associated with the NP in accordance with the *jellium* approximation. We recall that the NPs became negatively charged by releasing positive sodium ions. Hence, electroneutrality in the bulk of the suspension is a product of the negative charge on the NPs and the excess positive charge of ions due to the larger concentration of cations compared to the concentration of anions, *q*_1_ *n*_1_^*b*^ + *q*_2_ *n*_2_^*b*^. The background charge density by the NPs is then −(*q*_1_ *n*_1_^*b*^ + *q*_2_ *n*_2_^*b*^). Including the interaction energy between ions and the background charge to the EDL contribution to the grand potential adds the third term on the r.h.s. of the grand potential

26 $$\begin{array}{l} \Omega_{EDL}\left[n_{1},n_{2}\right]=k_{B}T\sum_{i=1}^{2}\int drn_{i}\left(r\right)\left[\ln\left(d_{NP}^{3}n_{i}\left(r\right)\right)-1\right]\\ +\frac{1}{2}\sum_{i=1}^{2}\sum_{j=1}^{2}\iint drdr^{\prime }n_{i}\left(r\right)n_{j}\left(r^{\prime }\right)\frac{q_{i}q_{j}}{4\pi \epsilon \left|r-r^{\prime }\right|}\\ -\sum_{i=1}^{2}\iint drdr^{\prime }q_{i}n_{i}\left(r\right)\frac{q_{1}n_{1}^{b}+q_{2}n_{2}^{b}}{4\pi \epsilon \left|r-r^{\prime }\right|}+\sum_{i=1}^{2}\int drn_{i}\left(r\right)\left[v_{i}\left(r\right)-\mu_{i}\right] \end{array}$$

Under the Derjaguin approximation, thus in a planar geometry, we define the steric potential of the hard walls on ion *i* of diameter, *d*_*i*_, as

27 $$v_{i}^{hw}\left(z\right)=\{\begin{array}{ll} \infty & \text{if}\;z<\frac{d_{i}}{2}\text{or}z>h-\frac{d_{i}}{2}\\ 0 & \text{if}\;\frac{d_{i}}{2}<z<h-\frac{d_{i}}{2} \end{array}$$

The Euler–Lagrange equations obtained from eq 26 in the planar geometry, where densities vary along the *z* coordinate, are

28 $$\frac{\delta \Omega_{EDL}}{\delta n_{i}\left(z\right)}=k_{B}T\;\ln\left(\frac{n_{i}\left(z\right)}{n_{i}^{b}}\right)+q_{i}\psi \left(z\right)=0,i=1,2,$$

where we used the chemical potentials $\mu_{i}=k_{B}T\;\ln\left(d_{NP}^{3}n_{i}^{b}\right)$ , since in the bulk *v*_*i*_(***r***) = 0 and the local charge is null. The electrostatic potential is $\psi \left(z\right)=\psi_{ext}\left(z\right)+\int dr^{\prime }\left[\sum_{j}\frac{q_{j}n_{j}\left(z^{\prime }\right)}{4\pi \epsilon \left|r-r^{\prime }\right|}-\frac{q_{1}n_{1}^{b}+q_{2}n_{2}^{b}}{4\pi \epsilon \left|r-r^{\prime }\right|}\right]$ . The hard-wall term *v*_*i*_^hw^ introduces the constraint that particles are confined within a distance of *d*_*i*_/2 from the walls.

With the current functional we retrieve the classic Boltzmann distributions, $n_{i}\left(z\right)=n_{i}^{b}e^{-q_{i}\psi \left(z\right)/k_{B}T}$ . However, the electrostatic potential, ψ­(*z*), accounts for the background charge by the NPs and follows the Poisson equation

29 $$\epsilon \frac{d^{2}\psi \left(z\right)}{dz^{2}}=-\left[q_{1}n_{1}\left(z\right)+q_{2}n_{2}\left(z\right)-\left(q_{1}n_{1}^{b}+q_{2}n_{2}^{b}\right)\right]$$

augmented by the introduction of the term −(*q*_1_ *n*_1_^*b*^ + *q*_2_ *n*_2_^*b*^), namely the mean NP charge density in the *jellium* approximation. We thus obtain an augmented Poisson–Boltzmann (PB) equation

30 $$\frac{d^{2}\psi \left(z\right)}{dz^{2}}=-\frac{e}{\epsilon }\left[n_{1}^{b}e^{-e\psi \left(z\right)/k_{B}T}-n_{2}^{b}e^{e\psi \left(z\right)/k_{B}T}-\left(n_{1}^{b}-n_{2}^{b}\right)\right]$$

where we used *q*_1_ = – *q*_2_ = *e* for the charges of the sodium and chloride ions, *e* being the elementary charge. This equation is solved for ψ­(*z*), where *n*_1_(*z*) and *n*_2_(*z*) are determined as explained in Supporting Information.

Using the Derjaguin approximation, the EDL interaction between the confining surfaces of the colloidal probe and flat surface is calculated from the osmotic pressure of the ions at the midplane, *z* = *h*/2, between two corresponding flat confining surfaces, where the electric field vanishes by symmetry. The excess EDL pressure in the *jellium* approximation reads

31 $$P_{EDL}\left(h\right)={\left\{k_{B}T\left[n_{1}\left(z\right)-n_{1}^{b}+n_{2}\left(z\right)-n_{2}^{b}\right]+\left(q_{1}n_{1}^{b}+q_{2}n_{2}^{b}\right)\psi \left(z\right)\right\}}_{z=h/2}$$

See Supporting Information for the proof of this equation. The first term (between square brackets) on the r.h.s. is classic and represents the excess osmotic pressure of the ions. The second term is the product of the opposite of the *jellium* charge and the electrostatic potential. The entropy (osmotic pressure) associated with the NPs is absent from this equation since under the *jellium* approximation their contribution is manifested solely as spatially even background charge similar to the one in the bulk of solution. It is accounted for in Ω_NP._ The pressure is then integrated from infinite separation, *h* → ∞, to obtain the interaction energy per unit area, *W*_EDL_(*h*), at a finite value of *h*. The interaction force between the flat substrate and the spherical probe normalized by the radius of the latter is given by the Derjaguin approximation: *F*_EDL_(*h*)/*R* = 2π*W*_EDL_(*h*).

Details of all numerical computations are given in the Supporting Information.

## Results and Discussion

We now use the experimental results subject to NP volume fractions ϕ = 0.0061, 0.031 and 0.065, and added NaCl salt concentrations *c*(NaCl) = 0.1, 1, and 5 mM^58^ as a reference to validate the different theoretical strategies given above in a real scenario. We note here and highlight in some of the figures to follow that an implication of using the effective diameter of NPs, *d*_NP,eff_, as compared to the nominal diameter of NPs, *d*_NP_, is that the effective volume fraction of NPs in the suspension, $\phi_{eff}=\phi \times {\left(d_{NP,eff}/d_{NP}\right)}^{3}$ , is greater than their nominal volume fraction, ϕ, reported in the experiment by an order of magnitude. Thus, NP suspensions that nominally appear dilute are effectively dense, reaching in some cases values near the packing limit of hard spheres.

### Hard-Sphere Models for the Nanoparticle Excluded Volume

In Figure 2, we show the AFM measurement of the force normalized by the probe’s radius^58^ in blue. The measured force between the silica colloidal probe and the flat silica surface across the suspension of silica NPs vanishes at large separations, *h* → ∞. Positive excess force relative to infinity corresponds to repulsion between the probe and flat surface, while negative force corresponds to attraction. The force oscillates at approximately fixed wavelength and increasing amplitude as the separation, *h*, decreases, due to interactions of the NP layers, whose density *n*_3_ also varies with *h*. As the colloidal probe approaches the flat surface, the approximately parallel layers of NPs next to the two surface begin to interact. When they are compressed so that the NP density, *n*_3_, calculated by our functional minimization, is on average higher than the bulk density, *n*_3_^*b*^, the measured force reaches a maximum. Further decreasing *h* requires the extraction of a layer of NPs from the gap between the probe and flat surface to the bulk of suspension. Following this event, the reduction in NP density, *n*_3_, below the bulk value, *n*_3_^*b*^, results in depletion and produces an attractive force. As *h* continues to decrease, layers that are closer to the surfaces, thus more packed (higher density peaks), are expelled, leading to the alternation of wider maxima and minima. Hence the force oscillation period gives the separation between the layers of NPs.

**Figure 2:**
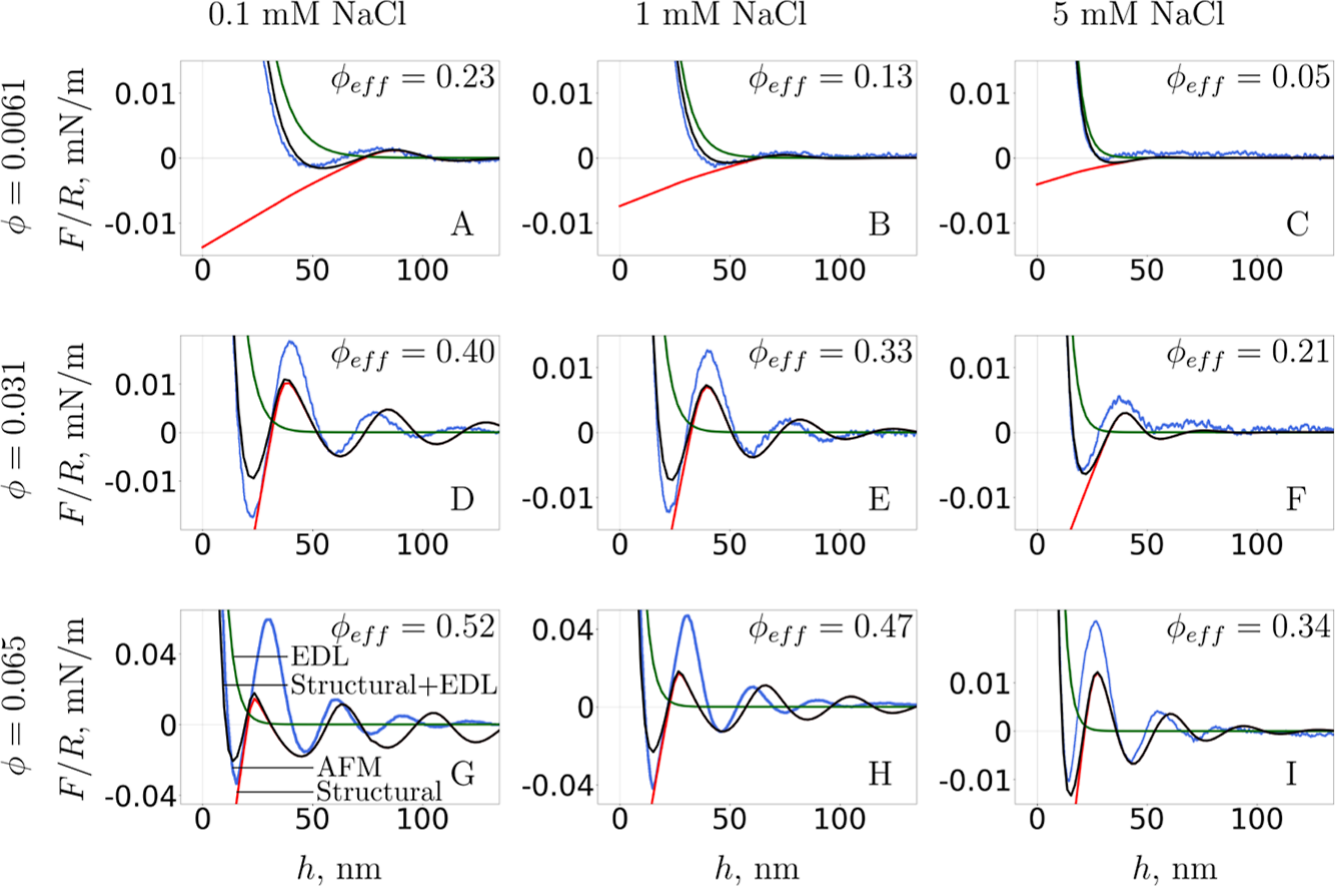
Separation variations of the oscillatory structural force (red) calculated using the Tarazona and Evans constant weight function and of the EDL force (green) calculated by solving the classic PB equation in the *jellium* approximation, where their superposition (black) is compared to AFM experiment (blue). The effective NP volume fraction, ϕ_eff_, accounts for the augmented NP diameter *d*_NP,eff_ and is given for each case.

Additionally, as *h* decreases, the measured force ceases to be symmetric about the zero force axis. The positive, repulsive, part of the oscillating force becomes greater in magnitude than the negative, attractive, component of the force. At sufficiently small separations, the measured force rises sharply and the experiment ends, as the probe comes in contact with the substrate. The increase in repulsion as *h* decreases is a product of the EDL repulsion between the probe and flat surface.

We compare in the figure the measured force to the theoretical result. We show in red the contribution to the interaction force from the NP excluded volume. The results are obtained using the simple uniform-weight theory, according to Tarazona and Evans, solving eqs 14 with [Disp-formula eq11] and 13 and using eq 4. We account for the interactions between NPs due to their hard (nominal) core diameter and inter-NP EDL interactions using the effective NP diameter in [Disp-formula eq22]. The green curve for contributions to the interaction force from EDL interactions between the confining surfaces is calculated by solving the PB equation with the *jellium* contribution, eqs 30 and 31. The black line is the superposition of the red and green curves, giving the full prediction of the theory.

The AFM data are correctly reproduced at the low NP concentrations, ϕ = 0.0061, where HS correlations are relevant at short-range. However, for ϕ ≥ 0.031, the theory produces a greater force oscillation wavelength than the experiment. The simplified weight function overestimates the range of NP correlations, artificially increasing the separation between layers of NPs and decreasing their average density, as also observed from comparison with simulations.^31^

Figure 3 compares the measured force curve with the theoretical force curves computed using the same effective NP diameter as before in eq 22, but using FMT as in eqs 15–17 for the contribution of the NP excluded volume (red curve). The green curves for contributions to the interaction force from EDL interactions between the confining surfaces are the same as the ones shown in the previous figure. The black line is the superposition of the red and green curves, giving the full prediction of the theory.

**Figure 3:**
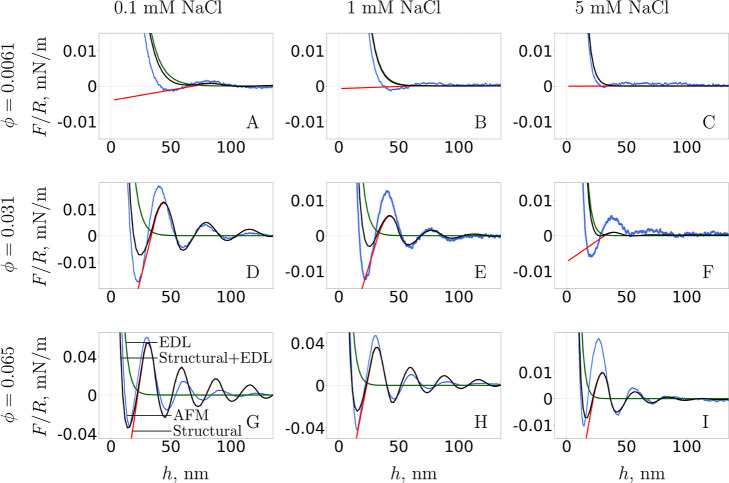
Separation variations of the oscillatory structural force (red) calculated usingFMT and of the EDL force (green) calculated by solving the classic PB equation in the *jellium* approximation, where their superposition (black) is compared to AFM experiment (blue).

Except for the maximum NP density (bottom row), the theoretical prediction for the force oscillation amplitude is smaller than the measured amplitude. This is especially true for ϕ = 0.0061, where, the oscillations almost disappear for the maximum concentration of added NaCl. Moreover, the first attractive minimum in the force is shallower than the second and certainly not as deep as in the experiment at low concentrations. However, FMT gives better agreement with theory for ϕ = 0.065. Most importantly, FMT captures better the period of oscillation in all cases, which can be immediately seen from comparison with the experimental force curves.

We have previously shown that the wavelength of the force oscillations, λ, corresponds to the interlayer separation of the same NPs ordered next to a planar hard wall perpendicularly to it, and only depends on the NP bulk density, *n*_3_^*b*^, according to a simple inverse cubic root law with a numerical prefactor γ: $\lambda =\gamma {\left(n_{3}^{b}\right)}^{-1/3}$ .^60^ This law was found to describe this specific measurement^58^ and appears to be universal, as it was observed in other experiments^28,53^ and simulations^26,27^ on charged particles. However, at high NP concentration the wavelength from the constant-weight DFT is greater than its prediction and varies with the amount of added salt. In Figure 4A we report the wavelengths extracted by fitting the force curves with a harmonic function and illustrate the discrepancies from the expected behavior. The latter is shown as black lines representing the scaling law $\lambda =\gamma {\left(n_{3}^{b}\right)}^{-1/3}$ , where the prefactor γ is between^60^ 0.89 and 0.92. Two out of three theoretical wavelengths at the intermediate concentration lie above the black lines and all are different from each other, indicating that they vary at different salt concentrations, which are represented by different colors, for the same *n*_3_^*b*^. For the highest concentration, ϕ = 0.065, the theory significantly overestimates the wavelength of the oscillations in all three cases. On the contrary, Figure 4B shows the wavelengths extracted from the FMT solution. These are extremely well represented by the aforementioned inverse cubic root law, as illustrated in Figure 4B, indicating that the theory better captures this well documented law.

**Figure 4:**
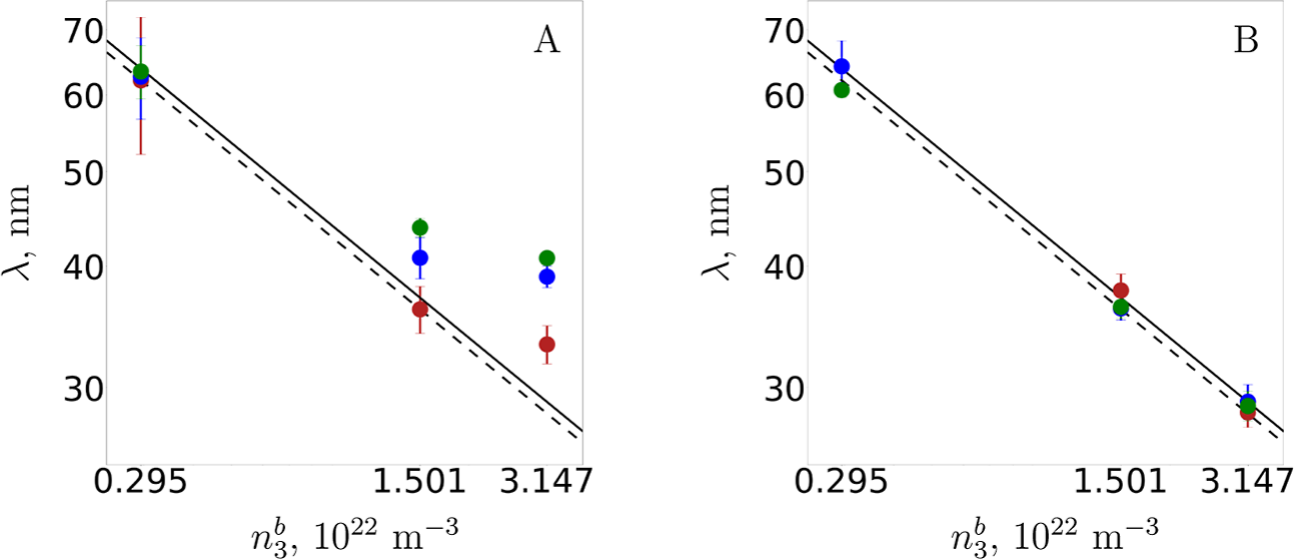
Oscillation wavelength λ extracted by fitting the force curves with $F/R=Ae^{-h/\xi }\;\cos\left[\left(2\pi /\lambda \right)h+\theta \right]$ as a function of the NP bulk density *n*_3_^*b*^ for three salt concentrations: 0.1 mM (green), 1 mM (blue), 5 mM (red) NaCl. The three NP densities on the horizontal axis correspond to ϕ = 0.0061, 0.031, and 0.065 from left to tight. The solid line is the law $\lambda =0.916{\left(n_{3}^{b}\right)}^{-1/3}$ , the dashed line is the law $\lambda =0.891{\left(n_{3}^{b}\right)}^{-1/3}$ .^60^ In A we give theoretical results based on the constant weight function, and in B we give theoretical results based on FMT; the wavelength for ϕ = 0.0061, 5 mM NaCl could not be determined due to the extremely small oscillations, see Figure 3C.

Therefore, using the simple constant-weight functional to capture contributions to the measured interaction force from the excluded volume of NPs is suitable in the case of dilute NP suspensions, as it correctly reproduces the measured oscillation amplitude and wavelength. For dense NP suspensions, the agreement between theory and experiment may be reasonable, albeit the prediction for the force oscillation wavelength becomes less precise. However, using FMT to capture contributions to the measured interaction force from the excluded volume of NPs gives particularly good agreement with experiment for dense NP suspension. The correct wavelength is further preserved across all NP concentrations.

### Soft EDL Potential

We further study the system of charged NPs by considering the inter-NP pair potential as the superposition of the nominal hard core of the NPs and a soft EDL tail, as in eqs 18 and 19. We implement the grand potential and fundamental equation for the NP density distribution in eqs 23 and 24. We found the computation challenging for our problem and stable solely for small force oscillations, i.e., for low NP and high salt concentrations. Figure 5 shows the resulting oscillatory force (red) for the case ϕ = 0.0061, *c*(NaCl) = 5 mM calculated with this method in A, compared to the previous HS theory in B, equivalent to Figure 2C. The force oscillations in A are wide and evident; the first minimum is shallow, albeit comparable to the experiment.

**Figure 5:**
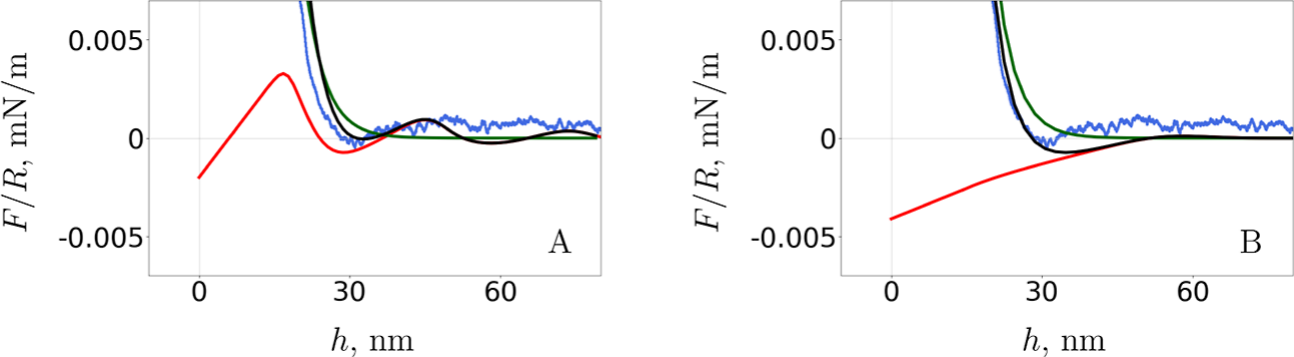
Separation variations of the oscillatory structural force (red) calculated usingthe soft EDL potential tail in A and using the effective HS model in B and of the EDL force (green) calculated by solving the classic PB equation. The superposition of both contributions to the theoretical force (black) is compared to AFM experiment (blue). NP concentration: ϕ = 0.0061, salt concentration: *c*(NaCl) = 5 mM.

This approach produces better agreement to experiment in the region around the first minimum than the previous HS approach, which accounts for the EDL interaction implicitly. However, the force oscillation wavelength is less precise. The reduced separation between consecutive repulsive force maxima is due to the reduction in the effective NP hard core size: the EDL repulsion between pairs of NPs is “soft”, meaning that it does not force the NPs to remain strictly outside of a volume shell. Therefore, the separation between the centers of NPs can become smaller than with the use of HSs with increased effective NP diameters. Overall, although it may be surprising, the effective diameter approach appears to be more suitable to describe the system of charged NPs than the explicit mean-field treatment of the long-range repulsive EDL interaction. Especially since the latter has clear limitations in reproducing the known behavior of the oscillation wavelength. It is however difficult to draw further conclusions on the soft-potential approach given that it is computationally demanding. The failure at reproducing real systems due to their complexity is the main flaw of the method. Further investigation of a simpler system than the one given here, which represent a physical experiment, might disclose whether this approach is viable for a wider range of system parameters and whether it better captures the physics of a corresponding NP system.

## Conclusions

We study different DFT strategies to model the structural and EDL forces experienced by two charged surfaces across a suspension of charged NPs in electrolyte. In this analysis we compare our theory to previous measurement,^58^ which differs from many previous studies that compared DFT to simulations of the same physics^5,37,39^ to verify the former. Reasonable agreement between theory and experiment unveils insight about the physics of the system that many times is not accessible to the experimentalist otherwise. Reproducing the qualitative nature and the magnitude of the experimental results, which our work achieved in the absence of fitting parameters, indicates that the theory captures the main physical mechanisms and the relevant geometrical features in the experiment. Hence, one may use the theory to better reveal the elusive nanometer-scale physics of the charged nanoparticles, the structure they form under confinement, and the complex electrostatic interactions therein, alongside understanding how they translate to the measured force at a micrometer scale in the experiment and the application to the stability of microparticles in charged nanoparticle suspensions.

In our work, we propose a new approach to model the long-range EDL interactions between charged NPs in electrolyte systems that enriches the discussion that commenced with the addition of one Debye length to the charged particle radius.^53,56,57^ The solution that we find most effective is to convert the interparticle hard-core and soft EDL tail interactions to a pure HS interaction with an effective diameter related to the second virial coefficient of the non-ideal gas theory, which accounts for the overall repulsion between the NPs The inter-NP EDL interaction increases the effective NP radius, although in a nontrivial way, accounting for both the ionic strength in the solution (related to the Debye length) and the NP surface charge. With this prescription, the results of the HS model reproduce the measurement well.

The alternative approach in which EDL interactions between NPs are treated separately with a dedicated soft repulsion term in the functional did not give definite results: we found the computations required to model the physical experiment challenging compared to the case of the effective HS model. We are able to obtain converged results using this method only in some specific cases, where the agreement with experiment is accurate in the proximity of the first force minimum, but the force oscillation wavelength is underestimated. Further investigation is needed to draw accurate conclusions.

In our analysis we compared two kinds of HS weighted-density functionals to describe the structural force: the constant-weight functional and the FMT. The former proved to be suitable for experiment at low NP density (ϕ_eff_ < 0.2), while giving good visual agreement with experiment at intermediate density (0.2 < ϕ_eff_ < 0.4), although showing some flaws in the wavelength prediction. The latter, on the contrary, predicts a wavelength in accordance to experiment and to the known scaling law (inverse cubic root scaling with the NP density). However, it tends to underestimate the amplitude of the measured force oscillations when these are narrow.

## Supplementary Material



## Notes

Basic DFT equations; formal description of the weighted density approximations compared in the work and the methods used for numerical solution; derivation of the double layer pressure expression (PDF)
