# Supplementary material for: Stability of Nano and Micro Particle Suspensions in Electrolyte Solutions: A Comparative Classical Density Functional Theory and Measurement of Force and Structure in Confined Complex Fluid
Source: J Phys Chem B. 2026 May 12;130(21):5387–98. doi: 10.1021/acs.jpcb.6c00681 (PMC13224178; doi:10.1021/acs.jpcb.6c00681)
Supplement: Supplementary file 1 [file jp6c00681_si_001.pdf]

On the Stability of Nano and Micro Particle Suspensions in Electrolyte Solutions: A Comparative Classical Density Functional Theory and Measurement of Force and Structure in Confined Complex Fluid

Simone Riva,[†] Michael Ludwig,[‡] Regine von Klitzing,[‡] and Ofer Manor[*,†]

†*Department of Chemical Engineering, Technion - Israel Institute of Technology, Haifa, 3200003, Israel*

‡*Department of Physics, Technische Universität Darmstadt, Darmstadt, 64289, Germany*

E-mail: manoro@technion.ac.il

Supporting Information

Classical density functional theory equations

We present the fundamentals of classical density functional theory (cDFT) an the equations relevant for a one-component liquid. The representation of the free energy in cDFT is not a function of individual degrees of freedom, i.e., positions and momenta, of all constituents of the system. Rather, it depends on a continuum variable, the local number density, $n(\boldsymbol{r})$, of molecules or particles that varies in space, which we represent by the spatial system

of coordinate $\boldsymbol{r}$. The free energy is sensible to spatial density variations and thus to the microscopic structure of a particulate liquid.

The grand potential of a liquid at constant temperature T, volume V, and chemical potentials μ_i, reads $\Omega = F - \sum_i \mu_i N_i = U - TS - \sum_i \mu_i N_i$, where U and F are the internal energy and Helmholtz free energy, respectively, S is the entropy and N_i is the number of molecules of species i.

The grand potential for a system of one type of liquid molecules or particles in a suspension with number density $n(\boldsymbol{r})$ may be expressed as a functional of the latter,[1]

$$\Omega[n] = \mathcal{F}[n] - \mu \int d\boldsymbol{r}\, n(\boldsymbol{r}) + \int d\boldsymbol{r}\, n(\boldsymbol{r}) V_{ext}(\boldsymbol{r}), \tag{S.1}$$

where the first term on the right hand side of the equation, $\mathcal{F}[n]$, is the intrinsic Helmholtz free energy, devoid of contributions from external potential fields, the second term removes the energy gain from adding particles in the system at a fixed chemical potential, and the last term accounts for the total energy contribution associated with the presence of the external potential $V_{ext}(\boldsymbol{r})$. Moreover, $\int d\boldsymbol{r}$ represents a volume integral of the system in question. The functional is variationally minimized to determine the equilibrium density, $n_{\mathrm{eq}}(\boldsymbol{r})$; the corresponding functional is the grand potential at equilibrium, Ω. The fundamental equation in the theory of inhomogeneous liquids is therefore:

$$\left.\frac{\delta \Omega[n]}{\delta n(\boldsymbol{r})}\right|_{n=n_{\mathrm{eq}}} = \left.\frac{\delta \mathcal{F}[n]}{\delta n(\boldsymbol{r})} - \mu + V_{ext}(\boldsymbol{r})\right|_{n=n_{\mathrm{eq}}} = 0, \qquad \Omega[n_{\mathrm{eq}}] = \Omega. \tag{S.2}$$

The chemical potential is the same in the confined particulate liquid and in the bulk reservoir and thus can be evaluated by applying this equation to the bulk suspension, where the constant bulk density is given by $n = n^b$ and the external potential vanishes, i.e., $V_{ext} = 0$, so that $\mu = \delta \mathcal{F}[n] / \delta n(\boldsymbol{r})|_{n=n^b}$.

In the case of a three-component system analyzed in our work, the grand potential is a functional of the densities of all three. Energy minimization by partial functional derivatives

with respect to all number density functions, $n_1,\ n_2,\ n_3$, is an extension of the fundamental derivative in (S.2) and yields Eq. (5) in the article text.

Weighted density approximations

Here we derive the three weighted density approximations we used for the contribution of the NPs to the grand potential functional, Ω_{NP}, and illustrate in detail the theories they are extracted from.

Constant weight

The constant weight function is the simplest weight function one can use. It was first introduced by Tarazona and Evans.[2] They focused on preserving short-range correlations, which are relevant in packing of a HS fluid near a hard wall, since the only inter-particle interactions are their excluded volume effects, rather than reproducing the whole correlation function. For this purpose, they used the expression in Eq. (14) in the text. This weight function was chosen empirically so that its range resembles the period of density oscillations and proved to be effective at least at low concentrations of NPs.

By solving Eq. (5) in the text iteratively with the prescriptions of Eq. (14) for w and Eq. (13) for Ψ and with the external potential of a hard wall,

$$V_{ext}(z) = \begin{cases} \infty & \text{if } z \leq \frac{d_{\mathrm{NP}}}{2} \\ 0 & \text{if } z > \frac{d_{\mathrm{NP}}}{2} \end{cases}, \tag{S.3}$$

they obtained the density profiles next to the wall. They further calculated the two-body direct correlation function,

$$c(\boldsymbol{r}, \boldsymbol{r}') = -\frac{1}{k_B T} \frac{\delta^2 \mathcal{F}_{ex}[n]}{\delta n_3(\boldsymbol{r}) \delta n_3(\boldsymbol{r}')}, \tag{S.4}$$

of a homogeneous system, achieving good agreement with the correlation function of the Percus-Yevick theory for volume fractions up to $\phi = 0.1$.

Density-dependent weight

The density-dependent weight is a functional developed semi-empirically in order to properly describe HS systems in a wider range of situations. In particular, it is aimed at extending the applicability of the previous functional to strongly structured suspensions, resolving the issue of the direct correlation function deviating from the Percus-Yevick solution. In fact, the simple constant weight overestimates the range of $c(r)$, introducing a negative tail for $d_{\mathrm{NP}} < r < 2d_{\mathrm{NP}}$. As a consequence, the distance between layers of NPs next to a hard wall predicted by the constant-weight theory is larger than in experiments and simulations, an effect that increases with concentration.

To improve the model, Tarazona introduced a weight function depending on the smoothed density $\bar{n}_3(\boldsymbol{r})$:[3]

$$\bar{n}_3(\boldsymbol{r}) \equiv \int d\boldsymbol{r}'\, n_3(\boldsymbol{r}') w(\boldsymbol{r} - \boldsymbol{r}', \bar{n}_3(\boldsymbol{r})). \tag{S.5}$$

This is an integral equation that defines $\bar{n}_3(\boldsymbol{r})$ once $w(\boldsymbol{r}, n)$ is chosen. Assuming that the weight is a power series of n and a radial function of $\boldsymbol{r}$, one can write

$$w(r, n) = w_0(r) + w_1(r)n + w_2(r)n^2 + ..., \tag{S.6}$$

where the series can be truncated at the second order and the following normalizations must hold:

$$\int d\boldsymbol{r}\, w_i(r) = \begin{cases} 1 & \text{if } i = 0 \\ 0 & \text{if } i = 1, 2, ... \end{cases} . \tag{S.7}$$

Obviously by retaining only the zero-order term, w_0, i.e., in the dilute limit, one must retrieve

the previous constant-weight approximation. Therefore, we take

$$w_0(r) = \begin{cases} \frac{3}{4\pi d_{\mathrm{NP}}^3} & \text{if } r \leq d_{\mathrm{NP}} \\ 0 & \text{if } r > d_{\mathrm{NP}} \end{cases} . \tag{S.8}$$

We now need to write explicitly the two following coefficients, $w_1(r)$ and $w_2(r)$, in order to achieve a higher-than-zero-order approximation in powers of the density, suitable for larger concentrations. For the first one, an analytical expression is found by comparing the linear terms in n in the cluster expansion of the direct correlation function for HSs and in $c(r)$ obtained with Eq. (S.4) from the functional we are considering with the current weight, see Tarazona[3]. This gives an integral equation for $w_1(r)$ depending on $w_0(r)$, which is known, that can be solved numerically. The following fitted polynomial and oscillating approximation is also available:

$$w_1(r) = \begin{cases} \frac{6}{\pi}\left[a_0 + a_1 \frac{r}{d_{\mathrm{NP}}} + a_2 \left(\frac{r}{d_{\mathrm{NP}}}\right)^2\right] & \text{if } r \leq d_{\mathrm{NP}} \\ \frac{6}{\pi}\left\{ c e^{-\beta_1\left(\frac{r}{d_{\mathrm{NP}}}-1\right)} \sin\left[\alpha\left(\frac{r}{d_{\mathrm{NP}}}-1\right)\right] + \right. & \\ \quad \left. + e^{-\beta_2\left(\frac{r}{d_{\mathrm{NP}}}-1\right)} \left[b_0 + b_1 \frac{r}{d_{\mathrm{NP}}} + b_2 \left(\frac{r}{d_{\mathrm{NP}}}\right)^2 + b_3 \left(\frac{r}{d_{\mathrm{NP}}}\right)^3\right]\right\} & \text{if } r > d_{\mathrm{NP}} \end{cases} , \tag{S.9}$$

with

$$\begin{aligned} &\alpha_0 = 0.90724, && \alpha_1 = -1.23717, && \alpha_2 = 0.21616, && \\ &c = -0.10244, && \alpha = 4.934, && \beta_1 = 3.5621, && \beta_2 = 12, \\ &b_0 = 35.134, && b_1 = -98.684, && b_2 = 92.693, && b_3 = -29.257. \end{aligned} \tag{S.10}$$

In practical application we truncate this function at $r = 2d_{\mathrm{NP}}$ with a correcting multiplying factor at $r \leq d_{\mathrm{NP}}$ to satisfy the normalization, Eq. (S.7). In particular, the inner part ($r \leq d_{\mathrm{NP}}$) needs to be multiplied by a prefactor of 1.302.

Tarazona found that the first-order approximation of $w(r,n)$ in n gives good results for the correlation function up to $\phi = 0.3$. If one wants to improve even more the second-order term is needed. This cannot be calculated exactly, but was rather determined empirically by Tarazona as

$$w_2(r) = \begin{cases} \frac{5}{4\pi}\left(\frac{6}{\pi}\right)^2\left[6 - 12\frac{r}{d_{\mathrm{NP}}} + 5\left(\frac{r}{d_{\mathrm{NP}}}\right)^2\right] & \text{if } r \le d_{\mathrm{NP}} \\ 0 & \text{if } r > d_{\mathrm{NP}} \end{cases}. \tag{S.11}$$

By adding this term, agreement with the Percus-Yevick correlation is obtained up to $\phi = 0.4$.

The free-energy minimizing equation reads,

$$\begin{aligned}\frac{\delta\Omega_{\mathrm{NP}}}{\delta n_3(z)} =& k_B T \ln\left(d_{\mathrm{NP}}^3 n_3(z)\right) + \Psi(\bar{n}_3(z)) + \int_0^h dz'\, n_3(z')\Psi'(\bar{n}_3(z'))\frac{Q(|z-z'|, \bar{n}_3(z'))}{1 - \bar{n}_1(z') - 2\bar{n}_2(z')\bar{n}_3(z')} \\ &- \mu = 0,\end{aligned} \tag{S.12}$$

with the smoothed densities $\bar{n}_i(\boldsymbol{r}) \equiv \int d\boldsymbol{r}'\, n_3(\boldsymbol{r}')w_i(|\boldsymbol{r} - \boldsymbol{r}'|)$ for $i = 0,\ 1,\ 2$ and the integral of the weight function over the transversal plane $x - y$, $Q(z,n) \equiv \int_0^\infty d\rho\, 2\pi\rho\, w(\sqrt{\rho^2 + z^2}, n)$. The smoothed densities $\bar{n}_i(\boldsymbol{r})$ also enable a simple calculation of the average density $\bar{n}_3(\boldsymbol{r})$, since truncating Eq. (S.6) at the second order, we obtain from the definition, Eq. (S.5),

$$\bar{n}_3(\boldsymbol{r}) = \bar{n}_0(\boldsymbol{r}) + \bar{n}_1(\boldsymbol{r})\bar{n}_3(\boldsymbol{r}) + \bar{n}_2(\boldsymbol{r})\bar{n}^2(\boldsymbol{r}). \tag{S.13}$$

This is a second-order equation, whose solution is

$$\bar{n}_3(\boldsymbol{r}) = \frac{2\bar{n}_0(\boldsymbol{r})}{1 - \bar{n}_1(\boldsymbol{r}) + \sqrt{[1 - \bar{n}_1(\boldsymbol{r})]^2 - 4\bar{n}_0(\boldsymbol{r})\bar{n}_2(\boldsymbol{r})}}. \tag{S.14}$$

Fundamental measure theory

Rosenfeld first established the fundamental measure theory (FMT)[4,5] to describe inhomogeneous hard sphere mixtures through a density functional whose pair direct correlation function is compatible with the Percus-Yevick theory. Our study involves all spheres of the same size rather than a mixture, so will the following equations. The starting point is the virial expansion of the excess free-energy of hard spheres, whose lowest-order approximation is given by

$$\frac{\mathcal{F}_{ex}}{k_B T V} = B_2 n_3^2 + ... \tag{S.15}$$

The virial coefficient $B_2 = \frac{2}{3}\pi d_{\mathrm{NP}}^3 = \frac{1}{2}\int d\boldsymbol{r}\, \Theta(d_{\mathrm{NP}} - |\boldsymbol{r}|)$ is the measure of half the volume excluded by the size of a couple of spheres, where Θ is the Heaviside theta function and corresponds to the Mayer function of hard spheres. In a non-local (weighted) functional formulation the previous equation translates in the dilute limit to

$$\frac{\mathcal{F}_{ex}[n_3]}{k_B T} = \frac{1}{2}\int d\boldsymbol{r} \int d\boldsymbol{r'}\, n_3(\boldsymbol{r}) n_3(\boldsymbol{r'}) \Theta(d_{\mathrm{NP}} - |\boldsymbol{r} - \boldsymbol{r'}|) + ... \tag{S.16}$$

The functional derivative now yields

$$\frac{\delta \mathcal{F}_{ex}[n_3]/k_B T}{\delta n_3(\boldsymbol{r})} = \int d\boldsymbol{r'}\, n_3(\boldsymbol{r'}) \Theta(d_{\mathrm{NP}} - |\boldsymbol{r} - \boldsymbol{r'}|) + ... = \frac{4}{3}\pi d_{\mathrm{NP}}^3 \bar{n}_3(\boldsymbol{r}) + ..., \tag{S.17}$$

where the average density $\bar{n}_3(\boldsymbol{r})$, is weighted using the previous constant weight. At this point the excess free energy can be written as a function of the weighted density, $\mathcal{F}_{ex}[n_3] = \int d\boldsymbol{r}\, n_3(\boldsymbol{r}) \Psi(\bar{n}_3(\boldsymbol{r}))$, for example with the Carnahan-Starling function, Eq. (13) in the text. This approach thus retrieves the previous approximation at low densities. However, Rosenfeld aimed at describing the problem in terms of geometrical properties of the individual spheres, rather than pairs. This is done in order to develop a theory for mixtures, but also allows to extend it more readily to higher orders in density, thus more-than-two bodies interactions, making it suitable for concentrated suspensions. He found a deconvolution of

the pair exclusion function, $\Theta(d_{\mathrm{NP}} - |\boldsymbol{r} - \boldsymbol{r}'|)$, (Mayer function of hard spheres) into the fundamental measures of one sphere in different dimensions, i.e., its volume, surface area and radius. For this purpose a single weight function is not sufficient, but six weight functions are used to account for the many-particle nature of the problem. Four scalar weights are the volume densities of the three aforementioned measures plus a scalar; the remaining two weight functions are vectorial and account for the direction of the sphere's surface and radius.

The six weight functions are

$$\begin{aligned}
\omega^{(0)}(\boldsymbol{r}) &= \frac{\delta\left(d_{\mathrm{NP}}/2 - |\boldsymbol{r}|\right)}{\pi d_{\mathrm{NP}}^2}, \\
\omega^{(1)}(\boldsymbol{r}) &= \frac{\delta\left(d_{\mathrm{NP}}/2 - |\boldsymbol{r}|\right)}{2\pi d_{\mathrm{NP}}}, \\
\omega^{(2)}(\boldsymbol{r}) &= \delta\left(d_{\mathrm{NP}}/2 - |\boldsymbol{r}|\right), \\
\omega^{(3)}(\boldsymbol{r}) &= \Theta\left(d_{\mathrm{NP}}/2 - |\boldsymbol{r}|\right), \\
\boldsymbol{\omega}^{(1)}(\boldsymbol{r}) &= \frac{\boldsymbol{r}}{|\boldsymbol{r}|}\omega^{(1)}(\boldsymbol{r}), \\
\boldsymbol{\omega}^{(2)}(\boldsymbol{r}) &= \frac{\boldsymbol{r}}{|\boldsymbol{r}|}\omega^{(2)}(\boldsymbol{r}),
\end{aligned} \tag{S.18}$$

where δ the Dirac delta function. The integral $\int d\boldsymbol{r}\, \omega^{(\alpha)}(\boldsymbol{r})$ gives the volume $\frac{1}{6}\pi d_{\mathrm{NP}}^3$ for $\alpha = 3$, the area πd_{NP}^2 for $\alpha = 2$, the radius $d_{\mathrm{NP}}/2$ for $\alpha = 1$, 1 for $\alpha = 0$ and 0 for the two vector weight functions. The decomposition of the pair exclusion function reads

$$\Theta(d_{\mathrm{NP}} - |\boldsymbol{r} - \boldsymbol{r}'|) = \omega_i^{(0)} \otimes \omega_j^{(3)} + \omega_i^{(3)} \otimes \omega_j^{(0)} + \omega_i^{(1)} \otimes \omega_j^{(2)} + \omega_i^{(2)} \otimes \omega_j^{(1)} - \boldsymbol{\omega}_i^{(1)} \otimes \boldsymbol{\omega}_j^{(2)} - \boldsymbol{\omega}_i^{(2)} \otimes \boldsymbol{\omega}_j^{(1)}. \tag{S.19}$$

Here we have denoted the convolutions

$$\omega_i^{(\alpha)} \otimes \omega_j^{(\beta)} \equiv \int d\boldsymbol{r}'\, \omega^{(\alpha)}(\boldsymbol{r}_i - \boldsymbol{r}')\omega^{(\beta)}(\boldsymbol{r}_j - \boldsymbol{r}'). \tag{S.20}$$

The decomposition is the sum of the convolutions of all pairs of weight functions whose

product has the dimensions of an inverse volume. There are 4 such pairs of scalar weights (0-3, 1-2, 2-1 and 3-0) and two pairs of vectorial weights (1-2 and 2-1), where a vectorial product is implied in the integral. Other deconvolutions are possible.[6] The weighted densities are labeled with α:

$$n^{(\alpha)}(\boldsymbol{r}) = \int d\boldsymbol{r}'\, n_3(\boldsymbol{r}')\omega^{(\alpha)}(\boldsymbol{r}-\boldsymbol{r}'). \tag{S.21}$$

With these definitions and using Eq. (S.19), Eq. (S.17) becomes

$$\begin{aligned}\frac{\delta\mathcal{F}_{ex}[n_3]/k_BT}{\delta n_3(\boldsymbol{r})} = \int d^3x\ \big[&n^{(3)}(\boldsymbol{x})\omega^{(0)}(\boldsymbol{r}-\boldsymbol{x}) + n^{(0)}(\boldsymbol{x})\omega^{(3)}(\boldsymbol{r}-\boldsymbol{x}) + n^{(2)}(\boldsymbol{x})\omega^{(1)}(\boldsymbol{r}-\boldsymbol{x})\\ &+n^{(1)}(\boldsymbol{x})\omega^{(2)}(\boldsymbol{r}-\boldsymbol{x}) - \boldsymbol{n}^{(2)}(\boldsymbol{x})\cdot\boldsymbol{\omega}^{(1)}(\boldsymbol{r}-\boldsymbol{x}) - \boldsymbol{n}^{(1)}(\boldsymbol{x})\cdot\boldsymbol{\omega}^{(2)}(\boldsymbol{r}-\boldsymbol{x})\big] + ...,\end{aligned} \tag{S.22}$$

with the sum containing again the same six pairs. This equation is related to the pair direct correlation function in the dilute limit. The expression for $\mathcal{F}_{ex}[n]$ is straightforward.[1]

This is, however, only the first order in the expansion. Now Rosenfeld derived an expression for the functional that approximates the expansion to higher orders, thus extrapolating the free-energy to higher densities while ensuring this limit at low densities. Assuming again an excess free energy functional that is a function of the six weighted densities of the form $\mathcal{F}_{ex}[n_3] = \int d\boldsymbol{r}\, \Phi(\{n^{(\alpha)}(\boldsymbol{r})\})$, he showed how the function $\Phi(\{n^{(\alpha)}(\boldsymbol{r})\})$ can be uniquely determined. He used all the products of weighted functions having the dimensions of a volume, ($n^{(0)}$, $n^{(1)}n^{(2)}$, $(n^{(2)})^3$, $\boldsymbol{n}^{(1)}\cdot\boldsymbol{n}^{(2)}$ and $n^{(2)}(\boldsymbol{n}^{(2)}\cdot\boldsymbol{n}^{(2)})$) as a basis for expanding Φ with coefficients that are functions of the dimensionless density $n^{(3)}$. The coefficients are determined requiring that Eq. (S.22) is satisfied in the limit $n \to 0$, as well as other thermodynamic relations, yielding

$$\begin{aligned}\Phi(\{n^{(\alpha)}(\boldsymbol{r})\}) = &k_BT\left[-n^{(0)}\ln(1-n^{(3)}) + \frac{n^{(1)}n^{(2)}}{1-n^{(3)}} + \frac{1}{24\pi}\frac{(n^{(2)})^3}{(1-n^{(3)})^2} - \frac{\boldsymbol{n}^{(1)}\cdot\boldsymbol{n}^{(2)}}{1-n^{(3)}}\right.\\ &\left.-\frac{1}{8\pi}\frac{n^{(2)}(\boldsymbol{n}^{(2)}\cdot\boldsymbol{n}^{(2)})}{(1-n^{(3)})^2}\right].\end{aligned} \tag{S.23}$$

The grand potential then becomes

$$\Omega_{\mathrm{NP}}[n_3] = \int d\boldsymbol{r}\, \left\{k_B T\, n_3(\boldsymbol{r}) \left[\ln\left(d_{\mathrm{NP}}^3 n_3(\boldsymbol{r})\right) - 1\right] + \Phi(\{n^{(\alpha)}(\boldsymbol{r})\})\right\} - \mu \int d\boldsymbol{r}\, n_3(\boldsymbol{r}). \quad \text{(S.24)}$$

Its functional derivative reads

$$\begin{aligned}\frac{\delta\Omega_{\mathrm{NP}}}{\delta n_3(\boldsymbol{r})} =& k_B T \ln\left(n_3(\boldsymbol{r})/n_3^b\right) + \sum_\alpha \int d\boldsymbol{r}' \left\{ \frac{\partial\Phi(\{n^{(\alpha)}(\boldsymbol{r}')\})}{\partial n^{(\alpha)}(\boldsymbol{r}')} - \left.\frac{\partial\Phi(\{n^{(\alpha)}(\boldsymbol{r}')\})}{\partial n^{(\alpha)}(\boldsymbol{r}')}\right|_{n_3=n_3^b} \right\} \frac{\delta n^{(\alpha)}(\boldsymbol{r}')}{\delta n_3(\boldsymbol{r})} \\ =& 0,\end{aligned} \quad \text{(S.25)}$$

and the chemical potential is

$$\mu = k_B T \ln\left(d_{\mathrm{NP}}^3 n_3^b\right) + \sum_\alpha \int d\boldsymbol{r}'\, \left.\frac{\partial\Phi(\{n^{(\alpha)}(\boldsymbol{r}')\})}{\partial n^{(\alpha)}(\boldsymbol{r}')}\right|_{n_3=n_3^b} \frac{\delta n^{(\alpha)}(\boldsymbol{r}')}{\delta n_3(\boldsymbol{r})}. \quad \text{(S.26)}$$

The derivatives $\frac{\partial\Phi(\{n^{(\alpha)}(\boldsymbol{r})\})}{\partial n^{(\alpha)}(\boldsymbol{r})}$ can be calculated analytically from Eq. (S.23), while $\frac{\delta n^{(\alpha)}(\boldsymbol{r}')}{\delta n_3(\boldsymbol{r})}$ are simply the weight functions $\omega^{(\alpha)}(\boldsymbol{r}' - \boldsymbol{r})$.

The results of the theory are exact to the low-density first-order approximation. The successive term, describing the overlapping of 3 or more spheres, thus relevant at higher densities, is approximate. For a more detailed analysis of FMT see the references.[1,7,8] The code that implements the theory, which we also used, is available.[9] It contains, among others, an option to represent a system confined by two plates, so that the geometry is planar and the effective equations become one-dimensional. The results of the computation are the NP density profiles and the grand potential energy per unit area, $\Omega(h)/A$, at different separations. The interaction energy is calculated via Eq. (6) and the force via the Derjaguin approximation.

Numerical methods

Solution of the NP problem

Eq. (16) in the article text is a nonlinear integral equation of the second kind and is solved numerically applying the Fixed Point Iteration (FPI) algorithm. This consists in isolating the unknown variable $n_3(z)$ on one side—in this case from the logarithmic term, since it is the only one containing just the non-weighted density—in order to write the equation in the form $n_3(z) = g[n_3(z)]$, and finding the fixed point of the function g. Here g is a non-local functional depending on n at all points z, meaning that the knowledge of the whole function $n_3(z)$ is required to calculate the new n at a specific point (or at least the values at a distance within the range of the weight function). The density at all points is calculated simultaneously along the iteration. The formula for the $(k+1)$-th iteration, staring from a density profile $n_3^k(z)$ and the corresponding weighted density $\bar{n}_3^k(z)$ and giving a new approximation $n_3^{k+1}(z)$, is then

$$\begin{aligned} n_3^{k+1}(z) =& n_3^k(z) + \alpha \left\{ \frac{1}{d_{\mathrm{NP}}^3} \exp\left[\frac{1}{k_B T} \left(-\Psi(\bar{n}_3^k(z)) \right.\right.\right. \\ & \left.\left.\left. - \frac{3}{4\pi d_{\mathrm{NP}}^3} \int_{|\boldsymbol{r}-\boldsymbol{r}'| \leq d_{\mathrm{NP}}} d\boldsymbol{r}' n_3^k(z') \Psi'(\bar{n}_3^k(z')) + \mu \right) \right] - n_3^k(z) \right\} . \end{aligned} \tag{S.27}$$

The iterative process starts from an initial guess, in our case a constant density $n_3^0(z) = n_3^b$, and calculates the corresponding weighted density profile $\bar{n}_3^0(z)$ and the new density for all z according to Eq. (16). The new approximation is taken as a linear superposition of the old approximation and this new solution, where the former has a relative weight $1-\alpha$ and the latter a weight α, with $0 < \alpha \leq 1$. Notice in fact that $\alpha = 1$ means that only the new solution is retained, while $\alpha = 0$ means that the $(k+1)$-th approximation is equal to the k-th, thus the algorithm is not progressing. The numerical parameter α is thus a mixing parameter damping the large density variation calculated with Eq. (16). It is needed for numerical reasons, as a non-damped iteration would easily diverge because of the exponential factor

and the tendency of Ψ to diverge at densities near the maximum packing. Thus, a trade-off needs to be found in choosing α, keeping in mind that lower α values more easily prevent divergence, but make convergence slower. Optimal values of α in our calculations were found between 0.06 and 0.12 and required about 40 iterations to achieve convergence. We calculate the 2-norm and ∞-norm of the difference between successive iterations to estimate the degree of convergence, requiring that the former is below 20% and the latter below 1% for the iterations to stop.

The free energy with the density-dependent weight is minimized with the same FPI algorithm. The main difference is the integral in Eq. (S.12), which replaces the one in Eq. (16). Its calculation requires averaging with three different weights, $w_0(r)$, $w_1(r)$ and $w_2(r)$. The integrals are carried out on different subsets of z points, as the zero and second-order weight functions are used to weight the density only within a sphere of radius d_{NP} around a reference point and the first-order up to a distance of $2d_{\mathrm{NP}}$, the range of truncated $w_1(r)$. In fact, $w_1(r)$, has in theory an infinite range, but in practice a cutoff at $r = 2d_{\mathrm{NP}}$ is applied, as mentioned, neglecting only an oscillating tail that has little effect. The total average $\bar{n}_3(\boldsymbol{r})$ is calculated from Eq. (S.14).

Numerically, in this case it is necessary to start from a smoother density distribution than in the zero-order approximation. Therefore, the starting density matches the maximum packing fractions—describing the hard walls—at the boundaries and decreases continuously to the bulk value at the center of the film. The iteration-damping parameter α needs to be lower in this case (0.002). The choice of an appropriate starting guess should reduce the number of iterations needed for convergence. Nonetheless, we were not able to achieve convergence with our resources.

Solution of the EDL problem

The Poisson-Botlzmann equation (32) in the text is a nonlinear equation for the electrostatic potential ψ that has to be solved with the boundary conditions $\psi(0) = \psi(h) = \psi_0 = -28.6$

mV, which is the measured surface potential at the two walls in the experiment we are modeling. The second-order ODE is solved with the shooting method: It is converted to a system of two first-order ODEs, one for the potential with known starting condition (the boundary value $\psi(0) = \psi_0$), and one for its derivative with unknown starting condition. The system is solved with a Runge-Kutta method as function of the unknown derivative at $z = 0$, ψ_0'. The correct ψ_0' is then determined by equating the resulting potential at the right boundary, a function of ψ_0', to the surface potential ψ_0. Once the potential profile, $\psi(z)$, is calculated, the ionic densities, $n_i(z)$, are obtained from the Boltzmann distribution

Derivation of the electrical double layer pressure in the *jellium* approximation

We have shown in the main text that under the *jellium* approximation the Poisson equation reads

$$\epsilon \frac{d^2\psi(z)}{dz^2} = -\left[q_1 n_1(z) + q_2 n_2(z) - \left(q_1 n_1^b + q_2 n_2^b\right)\right]. \tag{S.28}$$

This is trivial to prove, as the additional term compared to the traditional form, $-\left(q_1 n_1^b + q_2 n_2^b\right)$, is the uniform volume density of charge on the surface of the NPs, which is the opposite of the excess counterions charge density. By adding it, electroneutrality is satisfied in the bulk, where $\psi = \text{const} = 0$, and both sides of the equation vanish.

We will now derive the expression for the pressure experience by two charged surfaces interacting through their respective EDLs used in this work:

$$P_{\text{EDL}}(h) = \left\{k_B T[n_1(z) - n_1^b + n_2(z) - n_2^b] + (q_1 n_1^b + q_2 n_2^b)\psi(z)\right\}_{z=h/2}. \tag{S.29}$$

We particularly need to justify the last term. Let us start from the Gibbs-Duhem equation

of the electrolyte suspension at fixed temperature,

$$dP = \sum_{i=1}^{2} n_i d\mu_i. \tag{S.30}$$

Here the sum is over the two ionic species: Na^+ and Cl^-. Taking the ions' electrochemical potential $\mu_i(z) = \mu_{0,i} + q_i\psi(z) + k_B T \ln[d_{\mathrm{NP}}^3 n_i(z)]$, we differentiate the pressure along the transversal coordinate,

$$\frac{dP(z)}{dz} = \sum_i q_i n_i(z)\frac{d\psi(z)}{dz} + k_B T \sum_i \frac{dn_i(z)}{dz}. \tag{S.31}$$

Now using the Poisson equation we can substitute the total ion charge density, $\sum_i q_i n_i(z) = q_1 n_1(z) + q_2 n_2(z) = -\epsilon\frac{d^2\psi(z)}{dz^2} + q_1 n_1^b + q_2 n_2^b$. Here the inverse of the background charge, $q_1 n_1^b + q_2 n_2^b$, appears.

The derivative of the pressure thus becomes

$$\frac{dP(z)}{dz} = -\frac{\epsilon}{2}\frac{d}{dz}\left[\frac{d\psi(z)}{dz}\right]^2 + k_B T \sum_i \frac{dn_i(z)}{dz} + \left(q_1 n_1^b + q_2 n_2^b\right)\frac{d\psi(z)}{dz}. \tag{S.32}$$

Integrating from $z = \infty$ (bulk) to $z = h/2$ and using the fact that the potential vanishes far from the surfaces—$\psi(z)|_{z=\infty} = 0$ and $\left.\frac{d\psi(z)}{dz}\right|_{z=\infty} = 0$—and the symmetry of the electric field at $z = h/2$—$\left.\frac{d\psi(z)}{dz}\right|_{z=h/2} = 0$— we obtain the result, Eq. (S.29).

References

(1) Roth, R. Fundamental measure theory for hard-sphere mixtures: a review. *J. Phys.: Condens. Matter* **2010**, *22*, 063102–063119.

(2) Tarazona, P.; Evans, R. A simple density functional theory for inhomogeneous liquids. *Mol. Phys.* **1984**, *52*, 847–857.

(3) Tarazona, P. Free-energy density functional for hard spheres. *Phys. Rev. A* **1985**, *31*, 2672–2679.

(4) Rosenfeld, Y. Free-energy model for the inhomogeneous hard-sphere fluid mixture and density-functional theory of freezing. *Phys. Rev. Lett.* **1989**, *63*, 980–983.

(5) Rosenfeld, Y. Free energy model for inhomogeneous fluid mixtures: Yukawa-charged hard spheres, general interactions, and plasmas. *J. Chem. Phys.* **1993**, *98*, 8126–8148.

(6) Kierlik, E.; Rosinberg, M. L. Free-energy density functional for the inhomogeneous hard-sphere fluid: Application to interfacial adsorption. *Phys. Rev. A* **1990**, *42*, 3382–3387.

(7) Roth, R.; Evans, R.; Dietrich, S. Depletion potential in hard-sphere mixtures: Theory and applications. *Phys. Rev. E* **2000**, *62*, 5360–77.

(8) de Morais Sermoud, V.; de Freitas Gonçalves, A.; Barreto Jr., A. G.; Franco, L. F. M.; Tavares, F. W.; Castier, M. Classical density functional theory of confined fluids: From getting started to modern applications. *Fluid Phase Equilib.* **2024**, *586*, 114177–114203.

(9) Soares, E. A. PyFMT. 2022; `https://github.com/elvissoares/PyFMT`, Available at `https://github.com/elvissoares/PyFMT`.
